# The origin and radiation of the phosphoprotein phosphatase (PPP) enzymes of Eukaryotes

**DOI:** 10.1038/s41598-021-93206-8

**Published:** 2021-07-01

**Authors:** David Kerk, Jordan F. Mattice, Mario E. Valdés-Tresanco, Sergei Yu Noskov, Kenneth K.-S. Ng, Greg B. Moorhead

**Affiliations:** 1 Department of Biological Sciences, University of Calgary, 2500 University Dr. NW, Calgary, AB T2N 1N4 Canada; 2 Department of Chemistry and Biochemistry, University of Windsor, 401 Sunset Avenue, Windsor, ON N9B 3P4 Canada

**Keywords:** Enzymes, Proteins, Cellular signalling networks, Molecular evolution

## Abstract

Phosphoprotein phosphatase (PPP) enzymes are ubiquitous proteins involved in cellular signaling pathways and other functions. Here we have traced the origin of the PPP sequences of Eukaryotes and their radiation. Using a bacterial PPP Hidden Markov Model (HMM) we uncovered “BacterialPPP-Like” sequences in Archaea. A HMM derived from eukaryotic PPP enzymes revealed additional, unique sequences in Archaea and Bacteria that were more like the eukaryotic PPP enzymes then the bacterial PPPs. These sequences formed the basis of phylogenetic tree inference and sequence structural analysis allowing the history of these sequence types to be elucidated. Our phylogenetic tree data strongly suggest that eukaryotic PPPs ultimately arose from ancestors in the Asgard archaea. We have clarified the radiation of PPPs within Eukaryotes, substantially expanding the range of known organisms with PPP subtypes (Bsu1, PP7, PPEF/RdgC) previously thought to have a more restricted distribution. Surprisingly, sequences from the Methanosarcinaceae (Euryarchaeota) form a strongly supported sister group to eukaryotic PPPs in our phylogenetic analysis. This strongly suggests an intimate association between an Asgard ancestor and that of the Methanosarcinaceae. This is highly reminiscent of the syntrophic association recently demonstrated between the cultured Lokiarchaeal species *Prometheoarchaeum* and a methanogenic bacterial species.

## Introduction

**Figure 1:**
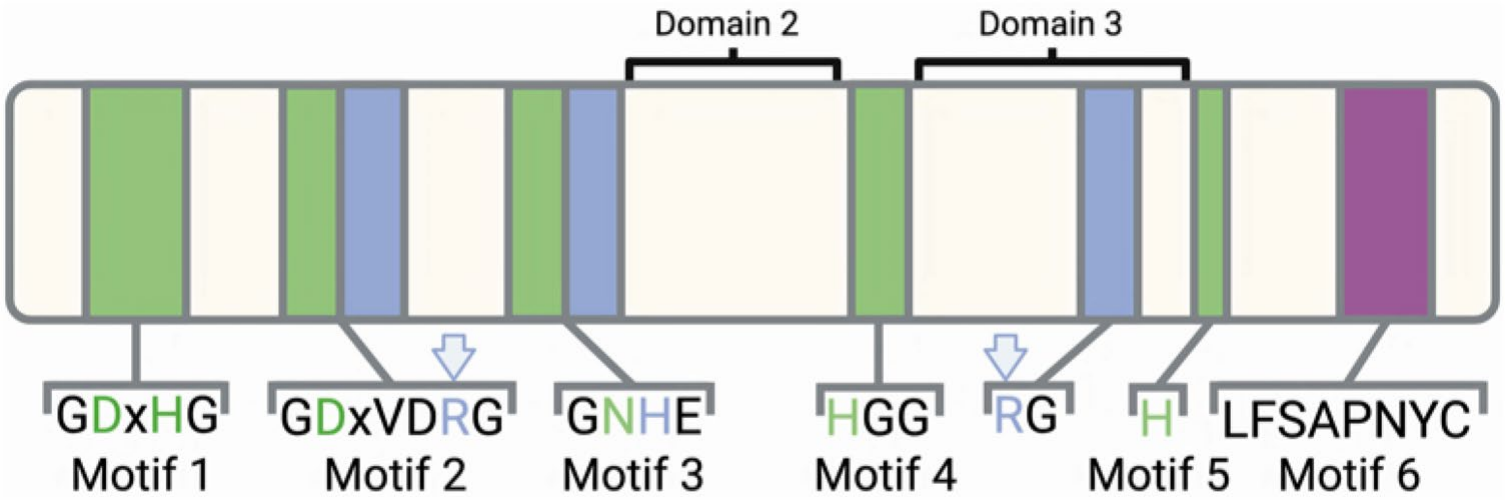
Conserved motifs and domains of the phosphoprotein phosphatases (PPP) of Eukaryotes. Shown is the representative motifs from human PP1α and location of domains 2 and 3. Metal binding residues are shown in green, while residues involved in catalysis (H) and the substrate interacting arginines (R) of the 2-Arginine Clamp are in blue and marked with arrows. Image was created using BioRender (biorender.com).

Mass spectrometry studies have established that more than three quarters of all human proteins are phosphorylated on one or more sites through a balance of activities of protein kinases and phosphatases^1–3^. It is also well established that protein phosphorylation is a common regulatory mechanism in all other Eukaryotes, bacteria and archaea. In a previous study^4^ we established that enzymes related to the major serine/threonine protein phosphatase family of Eukaryotes (the phospho-protein phosphatase or PPP family) is widely spread in bacteria and belongs to the broader metallophosphoesterase (MPE) (also called metallophosphatase (MPP)) superfamily. Using phylogenetics and molecular dynamics simulations we traced a likely evolutionary route from nuclease-like phosphodiesterase to a monoesterase with phospho-protein specificity in bacterial PPP enzymes^4^. All of the PPP enzymes have the same basic architecture with a series of conserved domains and motifs, as outlined in Fig. 1. Notable features are the highly conserved active site metal binding residues, a catalytic histidine (H) and the two arginines (R) that form the substrate binding 2-Arginine Clamp.

Utilizing this^4^ as a framework, we have now re-examined PPP evolutionary history. Combining HMM-derived archaeal and bacterial sequence datasets within structure-guided alignments, followed by phylogenetic tree inference, we trace the origin of eukaryotic PPPs to an archaeal ancestor related to the current Asgard superphylum. We analyze the evolutionary radiation of eukaryotic PPPs, and document previously unrecognized phylogenetic diversity, including novel phosphatase and regulatory domain architectures. Finally, we discuss the possible implications of PPP sequence evolution for the origin of eukaryotic cells.

## Results

### Search for PPP subtypes in bacteria and archaea

Previous reports^4,5^ described the evolution of bacterial PPPs from ancestral nuclease-like members of the MPE (metallophosphoesterase) superfamily^4^. We also described the important functional architecture of both bacterial and eukaryotic PPPs, including the presence of a “2-Arginine Clamp” in each sequence type which is important in phospho-substrate binding. Supplemental Figure S1 presents a summary alignment of an assortment of bacterial PPPs with human PP1, PP2A and PP5. The set of six classic motifs which characterize all PPPs is labeled, as are the conserved “clamp” Arg residues (also see Fig. 1).

In our previous report we also presented the sequence features of p-Ser/p-Thr and p-Tyr bacterial PPPs (i.e. sequences where representatives have been shown to be enzymatically active in vitro against substrates bearing either p-Ser/Thr or p-Tyr residues). To search for similar sequences within Archaea, we constructed an alignment of these bacterial sequences (presented as Supplemental Figure S2), produced an HMM, and searched the UniProt archaeal database. Candidate “BacterialPPP-Like” archaeal sequences were selected which shared all six conserved sequence motifs with the HMM. This produced a set of 119 sequences, which are summarized in Supplemental Table S1. An alignment containing these sequence groups is presented as Supplemental Figure S3. It can be readily observed that both the first (Motif 2) and second (Domain 3) Arg residues (arrows) of the “2-Arginine Clamp” of bacterial PPPs are conserved in the “BacterialPPP-Like” archaeal sequences. In addition, however, there is a loop unique to these archaeal sequences between Motif 5 and Motif 6, which contains another conserved Arg residue (arrow).

To inquire if additional PPP sequences exist which may be more related to eukaryotic PPP enzymes, an HMM was constructed from an alignment (presented as Supplemental Figure S4) of a large and diverse set of eukaryotic PPP sequences (206 sequences) and this was used to search the UniProt archaeal database. This produced a set of 224 sequences, which are summarized in Supplemental Table S2 and are desiginated “EukaryoticPPP-Like” archaeal sequences. A search with this HMM was also conducted of the UniProt bacterial database. This produced a set of 59 “EukaryoticPPP-Like” bacterial sequences, which are summarized in Supplemental Table S3.

There was a marked disparity in the distribution of the candidate sequences of various types. Within the “EukaryoticPPP-Like” sequences there were 224 archaeal candidates from a total database size of ~ 2.39E6 sequences, whereas there were approximately a quarter as many (59) bacterial candidates from a total database approximately 30 times larger (~ 74.56E6 sequences). Though there was a markedly enhanced number of archaeal as compared to bacterial “EukaryoticPPP-Like” candidates, their distribution within established archaeal groups was far from uniform. Although classically comprising only the Euryarchaeota and Crenarchaeota, a recent body of phylogenomics work, much of it utilizing previously unrecognized uncultured organisms, has markedly expanded archaeal phylogeny. A recent review of archaeal diversity^6^ includes the DPANNs (Diapherotrites, Parvarchaeota, Aenigmarchaeota, Nanohaloarchaeota, Nanoarchaeota); Euryarchaeota; Proteoarchaeota (comprising the previously proposed TACK supergroup (Thaumarchaeota, Aigarchaeota, Crenarchaeota, Korarchaeota)); and Asgard archaea (Lokiarchaeota, Thorarchaeota, Odinarchaeota, Heimdallarchaeota). A recent rooted tree of the Archaea^7^ places the DPANNs at the base, followed by Euryarchaeota, then the Proteoarchaeota. Interestingly, we recovered only three DPANN sequences in our extensive database HMM searching. In addition, the distribution of the other archaeal sequences was highly heterogeneous. Of the 20 euryarchaeal subgroups recently reported^6^, we obtained representatives from only six. Of the 10 Proteoarchaeota subgroups^6^, we obtained representatives of nine. Of the four Asgard subgroups reported^6^, we obtained sequences from three. Thus we observe a marked deficiency of the “EukaryoticPPP-Like” sequence type in basal archaeal groups. The “BacterialPPP-Like” archaeal sequences were almost entirely confined to the Euryarchaeota, the only exception being three sequences from uncultured archaea in environmental samples. Finally, the “EukaryoticPPP-Like” sequences of Bacteria were confined to a restricted taxonomic distribution, with 27/59 from Deltaproteobacteria (18 from Myxococcales), and seven from Firmicutes.

To explore the sequence relationship between these PPP/ PPP-like proteins a structure-guided alignment was then made containing bacterial PPPs, eukaryotic PPPs, “BacterialPPP-Like” archaeal sequences, “EukaryoticPPP-Like” archaeal sequences, and “EukaryoticPPP-Like” bacterial sequences (Supplemental Figure S5). As expected, the set of six sequence motifs (labeled) is conserved. The Arg residue in Motif 2 (down arrow) which constitutes the first part of the “2-Arginine Clamp” in both bacterial and eukaryotic PPPs is conserved in all sequences. The principal differences between these various sequence types are found in Domain 3, between Motif 4 and Motif 5. As noted previously (see Supplemental Figure S3) the second Arg residue of the “2-Arginine Clamp” implicated in enzymatic activity in bacterial PPPs (down arrow) is also observed in all the “BacterialPPP-Like” archaeal sequences. As also noted previously^4^ (see also Supplemental Figure S1), this residue is replaced by a structurally conserved Asp in eukaryotic PPPs (for example, Asp^208^ in human PP1α). In this alignment it can be seen that this Asp is also conserved in all the “EukaryoticPPP-Like” archaeal and bacterial sequences. Inspection also reveals that the predicted loop in Domain 3 (dashed underline) previously noted^4^ (see also Supplemental Figure S1) within eukaryotic PPPs is observed here in all the “EukaryoticPPP-Like” sequences (archaeal and bacterial) but not in the “BacterialPPP-Like” archaeal sequences. Near the end of this loop is an Arg residue (Arg^221^ in human PP1α) (up arrow), which was previously noted^4^ as the second residue of the “2-Arginine Clamp” important in phospho-substrate binding. This residue is conserved in all the eukaryotic PPPs in this alignment, and very nearly all sequences in a much larger eukaryotic PPP alignment (which will be presented later in Results as Supplemental Figure S12). This Arg residue is conserved in the great majority of the “EukaryoticPPP-Like” archaeal sequences, and in some of the “EukaryoticPPP-Like” bacterial sequences.

**Figure 2:**
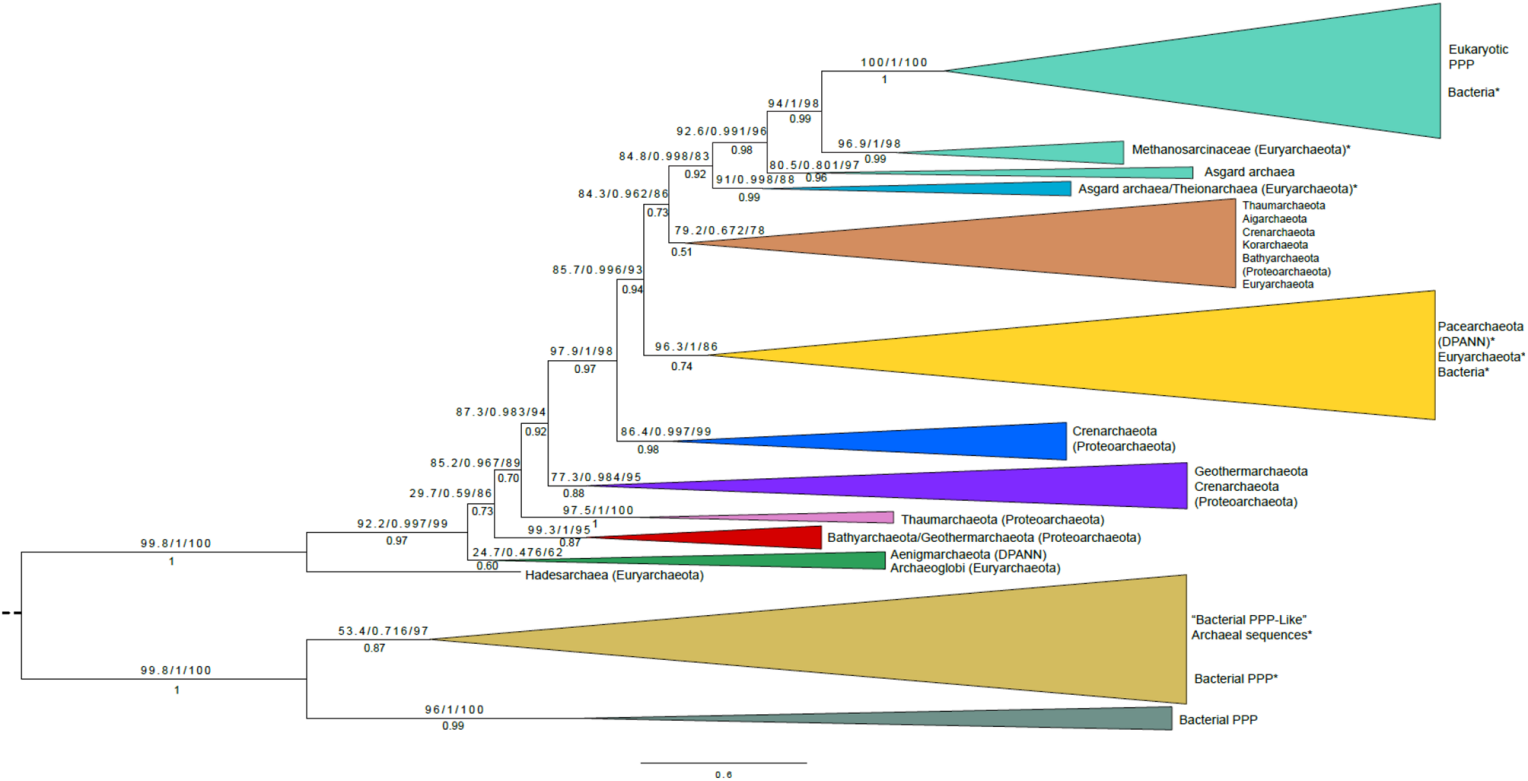
Evolution of PPP sequences in Archaea, Bacteria and Eukaryotes—simplified phylogenetic tree. Candidate PPP sequences were collected by Hidden Markov Model (HMM) based search methods, aligned, and phylogenetic trees inferred as detailed in “Methods”. Shown is a simplified representation of an orthogonal phylogram, with important sequence clusters depicted as cartoon branch expansions. The topology depicted is from the ML tree, but a virtually identical tree (identical in all shown branches) was obtained by Bayesian analysis. Support numbers above each branch represent the support in the ML tree (SH-aLRT/aBayes/UFBoot) (see “Methods”), whereas numbers below each branch represent the posterior probability in the Bayesian tree. The trees were inferred as unrooted, but the root (dashed line) was placed by separate BEAST analysis (data not shown). A detailed orthogonal phylogram is presented as Supplemental Figure S7, and a radial phylogram is presented as Fig. 3. In the trees, individual sequences or groups whose placement is likely to be due to lateral gene transfer (LGT) (see Text) have an asterisk. The alignment giving rise to these trees is presented as Supplemental Figure S6. The component candidate sequences are summarized in Supplemental Table S1 (archaeal candidates, “BacterialPPP-Like”), Supplemental Table S2 (archaeal candidates, “EukaryoticPPP-Like”), and Supplemental Table S3 (bacterial candidates, “EukaryoticPPP-Like”).

In order to facilitate better computational performance in phylogenetic tree inference methods [speed in Maximum Likelihood (ML), chain convergence in Bayesian], sequence clustering was used (see “Methods”) to reduce the dataset from 485 sequences in the alignment of Supplemental Figure S5 to 254 sequences. ML and Bayesian trees produced virtually identical topologies, with most clusters receiving high support. A simplified cartoon tree is presented as Fig. 2 (the edited alignment corresponding to this tree (and those to follow) is presented as Supplemental Figure S6).

**Figure 3:**
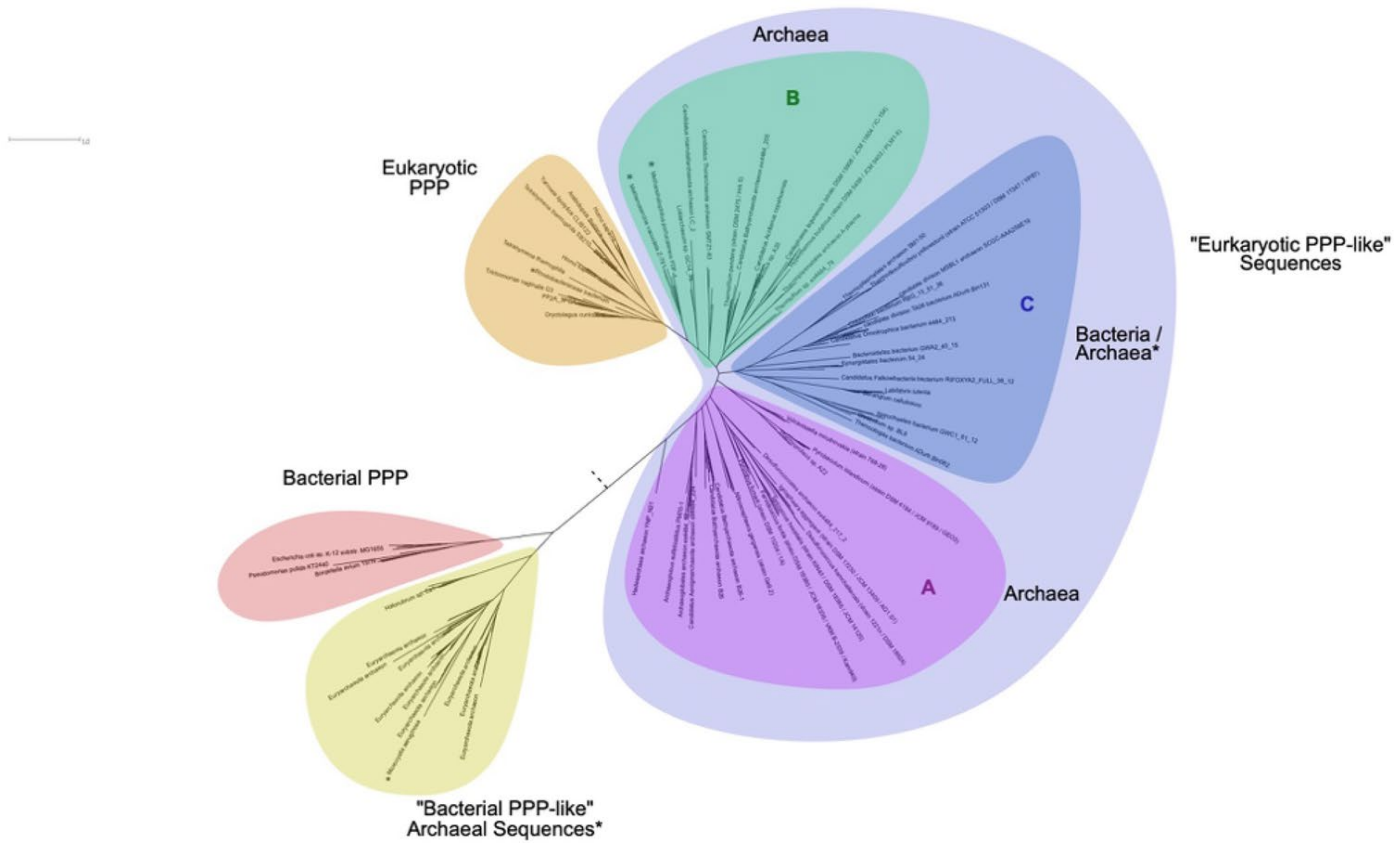
Evolution of PPP sequences in Archaea, Bacteria and Eukaryotes—radial phylogenetic tree. Candidate PPP sequences were collected by Hidden Markov Model (HMM) based search methods, aligned, and phylogenetic trees inferred as detailed in “Methods”. Shown is a radial phylogram. The topology depicted is from the Maximum Likelihood (ML) tree. The tree was inferred as unrooted, but the root (dashed line) was placed by separate BEAST analysis (data not shown). A simplified cartoon representation of an orthogonal phylogram is presented as Fig. 2, and a detailed orthogonal phylogram is presented as Supplemental Figure S7. In the trees, individual sequences or groups whose placement is likely to be due to lateral gene transfer (LGT) (see Text) have an asterisk. Tree regions “A”, “B”, and “C” are indicated (discussed in the Text). The alignment giving rise to these trees is presented as Supplemental Figure S6. The component candidate sequences are summarized in Supplemental Table S1 (archaeal candidates, “BacterialPPP-Like”), Supplemental Table S2 (archaeal candidates, “EukaryoticPPP-Like”), and Supplemental Table S3 (bacterial candidates, “EukaryoticPPP-Like”).

A detailed tree is presented as Supplemental Figure S7, and a simplified radial tree as Fig. 3.

In its overall topology (Fig. 3), the tree presents a “BacterialPPP-Like” wing (bacterial PPPs and Bacterial PPP-Like archaeal sequences), and a “EukaryoticPPP-Like” wing (eukaryotic PPPs and “Eukaryotic PPP-Like” archaeal and bacterial sequences), with the root lying between them. Though the ML and Bayesian trees were inferred as unrooted, the root (dashed line) was placed by separate BEAST analyses (data not shown). The “EukaryoticPPP-Like” wing can be further subdivided into three regions (“A”, “B”, and “C” (Fig. 3, Supplemental Figure S7).

Regions “A” and “B” consist exclusively of archaeal sequences. In contrast, region “C” contains a mixture of sequence clusters from both Archaea and Bacteria. This mixed sequence composition of region “C” strongly suggests the possibility of lateral gene transfer (LGT) between its two component organismal groups (i.e. Bacteria and Archaea). Furthermore the presence of this region, interposed between two regions of exclusively archaeal sequences, strongly suggests a distinct gene origin.

As noted previously, Euryarchaeota have a patchy representation in our dataset, with only six of the twenty currently described organismal groups being present. Their sequences are dispersed in regions “A”, “B”, and “C” of the tree (Fig. 3), rather than being clustered together. This strongly suggests that they arose at least in part through LGT. Region “A” of the tree contains a sequence from Hadesarchaea and a cluster from Archaeoglobi at its base, near the tree root. This suggests the entrance of “EukaryoticPPP-Like” sequences into these euryarchaeal groups. The remainder of region “A” consists of several sequence clusters from various groups of Proteoarchaeota. This suggests vertical inheritance from the basal Euryarchaeota. At the base of region “B” of the tree there is a large sequence cluster consisting of a basal Euryarchaeal group (Thermoplasmatales) followed by several Proteoarchaeota groups. This also suggests vertical inheritance from the Euryarchaeota to the Proteoarchaeota.

In the portion of segment “B” furthest from segment “C”, sequences from Asgard archaea appear in the position one would expect from further vertical inheritance (*i.e.,* distal to the root from various Proteoarchaeota groups). However, intermixed with them are groups of Euryarchaeota (Theionarchaea and Methanosarcinaceae) well separated from other Euryarchaeota in the tree. This suggests LGT rather than vertical inheritance as the source of these Euryarchaota sequences, from an ancestral Asgard source. Unexpectedly, the sequences from the Methanosarcinaceae are a sister-group to those from Eukaryotes, with high support, in both the ML and Bayesian trees.

### Structural insights into “EukaryoticPPP-Like” sequence evolution

We have previously generated a structural model for eukaryotic (human) PP1α complexed to a p-Ser peptide^4^. This led us to re-examine in more detail the possible impact of structural features on the evolution of “EukaryoticPPP-Like” sequences. As previously described^4^, the “2-Arginine Clamp” in this structure consists of Arg^96^ in Motif 2 plus Arg^221^ in a loop specific to “EukaryoticPPP-Like” sequences (see Supplemental Figure S1), with Arg^221^ being stabilized by Asp^208^ (residue numbering based on human PP1α). A re-examination of the large alignment of bacterial, archaeal and eukaryotic sequences in Supplemental Figure S5 shows that a “DP” residue pair is highly conserved at the entrance to this loop, as is an “RG” pair within the loop. This conservation is also evident in the large alignment of eukaryotic PPPs in Supplemental Figure S12. This suggests that the proline residue following the stabilizing Asp^208^ is important to its activity, perhaps because the unique structure of proline restricts the conformations available to the Asp side chain. The high degree of conservation of these two doublets also suggests that they were both important in establishing the functional activity of the “2-Arginine Clamp” in “EukaryoticPPP-Like” sequence evolution. This loop region has been isolated, and is presented as Supplemental Figure S8. Close inspection toward the bottom of the alignment reveals variant sequences which do not have both doublets. These come from sequence clusters in region “C” of the phylogentic trees of Fig. 3 and Supplemental Figure S7.

### Remodeling of the PPP carboxy terminus

**Figure 4:**
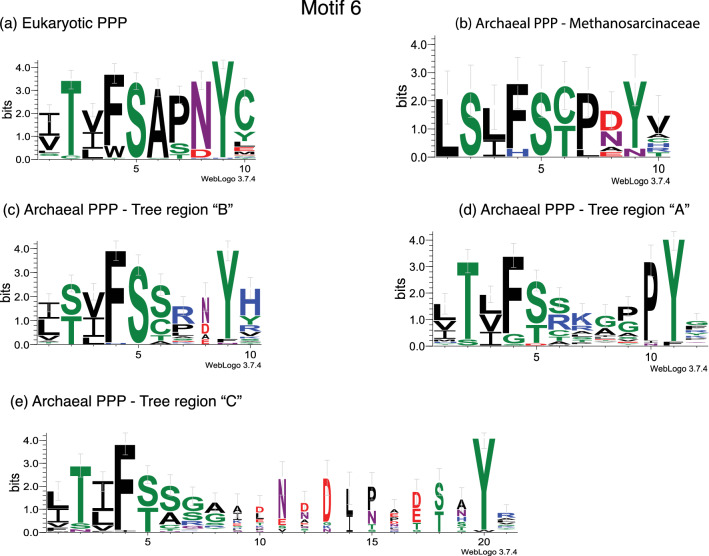
Residue conservation in Motif 6—graphical summary. Reference eukaryotic PPP sequences and candidate “EukaryoticPPP-Like” sequences from Archaea and Bacteria were collected, aligned, and phylogenetic trees constructed as detailed in “Methods”. Subalignments of the Motif 6 region were constructed as detailed in “Methods”. These were then converted into graphical summaries by the WebLogo3 server, as detailed in “Methods”. Each “stack” in the WebLogo (i.e. vertical set of characters) corresponds to a conventional alignment column. Conservation along the Y-axis of the WebLogo for each stack is given in units of “bits” of information. The frequency of occurrence of each character in the alignment column is represented by the height of that character in the WebLogo. The width of the stack corresponds to the number of gaps in the alignment column (i.e. columns with no gaps are widest, those with many gaps are narrowest). WebLogos were constructed from subalignments representing sequences in the major regions of the detailed phylogenetic tree of Supplemental Figure S7 and the radial phylogram of Fig. 3. The conventional alignments from which these WebLogo representations were constructed are presented as Supplemental Figure S9.

Additional inspection of the alignment in Supplemental Figure S5 indicates that the carboxy terminus (Motif 6) is a region of profound difference between “BacterialPPP-Like” and “EukaryoticPPP-Like” sequences. To examine this motif in “EukaryoticPPP-Like” sequences we gathered detailed subalignment data from various regions of the detailed phylogenetic tree presented in Supplemental Figure S7. Graphical summary data are presented as Fig. 4, while the underlying sequence alignment data is presented as Supplemental Figure S9.

It is readily apparent that the Motif 6 sequence from the Methanosarcinaceae is most similar to that of the eukaryotic PPPs, followed by that of region “B”, region “A” and region “C”. This ordered similarity recapitulates the general topology of the previously presented phylogenetic trees (Figs. 2, 3, Supplemental Figure S7), with regions “A” and “C” being deepest in the trees (i.e. closest to the root) and the Methanosarcinaceae a postulated sister group to the Eukaryotes. Part of Motif 6 is the canonical string SAPNY, prominent in PP1 and most other eukaryotic PPPs. It is clear that the Tyr residue (corresponding to Tyr^272^ of human PP1α) is well conserved in sequences from all regions of the tree. The early appearance and conservation of this residue suggests an important role. We examined the behavior of this residue in Molecular Dynamics (MD) simulations of our PP1:p-Ser peptide model as described in^4^. Tyr^272^ interacts by hydrogen bonding with Arg^96^ (the first residue of the “2-Arginine Clamp”), and also with an inserted water molecule completing the coordination shell of Metal 1. Finally, it is interesting to note that the final Cys residue in the motif in eukaryotic PPPs (Cys^273^ of human PP1α) is not shared by any of the archaeal sequence groups. This indicates that this may be one of the last structural variants of Motif 6 to evolve, after divergence of the eukaryotic sequences from their last common ancestor with Archaea. This residue is well known to be the site of covalent attachement of the algal toxin microcystin to PP1^8^.

### Radiation of eukaryotic PPP sequences

We next sought to investigate the evolutionary radiation of PPPs within Eukaryotes. We used our eukaryotic PPP HMM to search the UniProt eukaryotic sequence database using an iterative search strategy. As a proxy for testing eukaryotic PPP distribution we also constructed a set of protein databases from 45 species with completely sequenced genomes, and searched these with our HMM. As expected, we found none of our proxy set of 45 species lacked PP1 sequences (detected by the NCBI model cd07414) or the PP2A-PP4-PP6 family, suggesting a universal eukaryotic distribution. PP5s were found to be missing in the Microsporidia *Enterocytozoon bieneusi* and *Nosema ceranae*. However, this loss is probably secondary, as we found a PP5 in the microsporidian *Encephalitozoon intestinalis*. Similarly, we failed to find a PP5 in the green alga *Chlorella variabilis*, however we did find one in *Micromonas pusilla*. Thus PP5 also would appear to represent a universally distributed PPP type.

In contrast to the above universally distributed PPP types, other eukaryotic PPPs have previously been shown to have a more restricted distribution. Due to the expanding set of sequenced eukaryotic genomes, we revisited this question using our iterative HMM search based strategy. Candidate sequences (summarized in Supplemental Table S4) were aligned and subjected to phylogenetic tree inference. The results are summarized in Supplemental Figure S10 (Maximum-Likelihood tree) and Supplemental Figure S11 (rooted Bayesian tree). (The corresponding alignment is presented as Supplemental Figure S12). Bsu1s had previously been described in plants, green algae, and in a few alveolates (Ciliates and Apicomplexa)^9^. We found new examples in a greater variety of Apicomplexa, the Chromerida (*Chromera velia* and *Vitrella brassicaformis*), the dinoflagellate *Symbiodinium microadriaticum* and the alveolate *Perkinsus marinus*. All of these Bsu1s had the previously described Kelch repeat accessory domains. In addition, however, we found another set of Bsu1s that lack the Kelch repeat, but have EF Hands as accessory domains, including the apusozoan *Thecamonas trahens*, the choanoflagellates *Monosiga brevicollis* and *Salpingoeca rosetta*, the fungus *Basidiobolus meristosporus*, and the stramenopile *Nannochloropsis gaditana*. These data indicate that the Bsu1s are an ancient group with a much wider phylogenetic range than previously appreciated.

PP7s have been previously described in plants and green algae^10^. We found new examples in the red algae *Galdieria sulphuraria* and *Chondrus crispus*, and in the rhizarian *Plasmodiophora brassicae*. PP7s have been shown to possess a characteristic insertion within the phosphatase domain, which has been suggested to be autoinhibitory^11^. This insertion is shared by these new PP7 examples, as shown in the alignment in Supplemental Figure S13. In addition, we found a set of sequences from archamoebae of the genus *Entamoeba* which also contain this characteristic phosphatase domain insertion (see Supplemental Figure S13). These cluster outside the classic PP7 clade, and we have termed them “PP7-Like”. We found one very interesting sequence (A0A0M0JRT4), from the haptophyte *Chrysochromulina*, which appears to contain a divergent copy of the PP7 insertion (see Supplemental Figure S13). This sequence, rather than clustering with the PP7s, however, clusters with the PP5s, though with reduced support. This suggests that the PP5 group may have evolved from a PP7-like ancestor, and is consistent with the relative positions of the PP7 and PP5 groups in our phylogenetic trees of Supplemental Figures S10 and S11.

**Figure 5:**
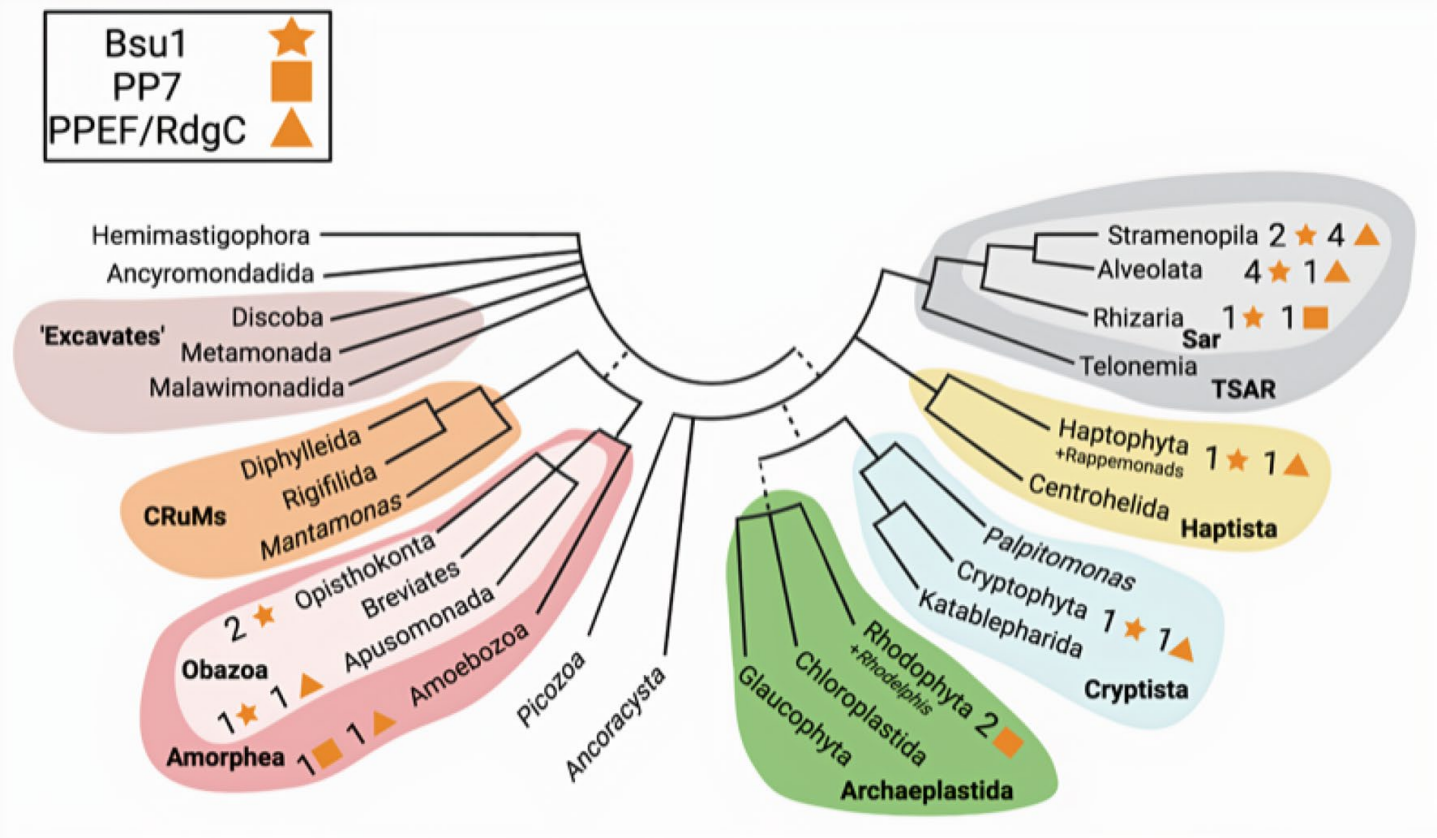
Eukaryotic PPP subtype sequences mapped onto the current Eukaryotic tree of life. Candidate novel sequences for eukaryotic PPP (phosphoprotein phosphatase) subtypes were collected from an iterative database search utilizing eukaryotic PPP HMMs as detailed in “Methods”. Candidates were validated by sequence alignment and phylogenetic tree inference, as detailed in “Methods”. The alignment of these validated sequences, together with reference eukaryotic PPPs, is presented as Supplemental Figure S12. The phylogenetic trees encompassing these sequences are presented as Supplemental Figure S10 (Maximum Likelihood) and Supplemental Figure S11 (rooted Bayesian). The Eukaryotic tree of life was modelled after the tree present in^12^. The number of newly identified sequences for Bsu1 (star), PP7 (square) and PPEF/RdgC (triangle) are indicated. Major organismal groups where the sequences are found is detail in Supplemental Figure S14. Image was created using BioRender (biorender.com).

Finally, PPEFs (protein phosphatases with EF-hand motifs) (also called RdgC for the *Drosophila* mutant Retinal Degeneration C) had been previously reported to have a wide distribution, including various Metazoa, Euglenozoa, Alveolates (including Apicomplexa and dinoflagellates), Oomycetes, and chlorophyte algae^10^. We found various new examples, expanding the above groups to include new species. For most of these sequences, the characteristic EF-hand-containing accessory domain was detected along with the phosphatase domain. The phylogenetic range of eukaryotic PPPs of the Bsu1, PP7, and PPEF/RdgC types is presented in Fig. 5 using the current Eukaryotic tree of life^12^ and detailed in Supplemental Figure S14 (modified with permission from reference^13^), indicating the contributions of the present report.

## Discussion

In a previous report^4^ we documented the origin of bacterial phosphoprotein phosphatases (PPP) from ancestral nuclease-like members of the metallophosphoesterase (MPE) superfamily. This article demonstrated, utilizing docking and molecular dynamics simulations with p-Ser/Thr peptides, that both bacterial PPPs (as exemplified by the phage lambda phosphatase [PDB:1G5B]) and eukaryotic PPPs (as ememplified by PP1 [PDB:3E7B]) bind substrate using functionally equivalent forms of a “2-Arginine Clamp”. This involves an upstream Arg in classic Motif 2 and a downstream Arg in Domain 3, between classic Motif 4 and Motif 5 (see Supplemental Figure S1). In this report we have extended this project to characterize the emergence of “EukaryoticPPP-Like” sequences in Bacteria, Archaea and Eukaryotes, and the subsequent radiation of PPP subtypes within Eukaryotes.

Our Hidden Markov Model (HMM) searches allowed us to identify candidates for “BacterialPPP-Like” sequences within the Archaea. These were very few in number, and almost entirely restricted in their distribution to the Euryarchaeota. These characteristics argue that these sequences were the subjects of lateral gene transfer (LGT) from Bacteria to Archaea, rather than arising through vertical inheritance from a common ancestor. This is supported by the finding that the sequence for PP1-cyano1, a p-Ser/p-Thr and p-Tyr PPP^4^ (active against p-Ser/Thr and p-Tyr substrates in vitro) from *Microcystis aeruginosa*, clusters together with the “BacteriaPPP-Like” archaeal sequences (Supplemental Figure S7, Fig. 3). The topology of the radial tree in Fig. 3 clearly shows that the archaeal “BacterialPPP-Like” sequences are segregated to their own wing. This suggests their lack of participation further in developments which led to eukaryotic PPPs.

The “EukaryoticPPP-Like” sequence set we identified by a HMM search in Archaea includes three members whose biochemistry has been characterized: PP1-arch (*Sulfolobus solfataricus*)^14^ (UniProt:Q55059), PP1-arch2 (*Methanosarcina thermophila*)^15,16^ (UniProt:O34200) and Py-PP1 (*Pyrodictium abyssi*)^17^ (UniProt:O08367). Each of these has been demonstrated to have p-Ser/Thr activity. Two of them (PP1-arch, PP1-arch2) have in addition been demonstrated, importantly, not to have p-Tyr activity. The specificity of eukaryotic PPPs for p-Ser/Thr and not p-Tyr is well established^18,19^. Our structure-guided alignment data (Supplemental Figure S1, Supplemental Figure S5) show that the eukaryotic PPPs and the “EukaryoticPPP-Like” sequences share the common substitution of Asp for the second Arg in the bacterial PPP “2-Arginine Clamp”, and an Arg residue within a specific loop that is highly conserved in eukaryotic PPP structures and sequences but that is not found in bacterial PPP sequences. In our previous report^4^ this Arg within the loop was also shown functionally to act as the second residue in the “2-Arginine Clamp” of human PP1. The biochemical properties of the investigated archaeal PPPs, the sequence conservation patterns evident in our current data, plus our previous demonstration of a distinct form of the “2-Arginine Clamp” for substrate binding in Eukaryotes, together strongly suggest that these structural and biochemical properties arose together in Archaea and were subsequently inherited in the PPPs of Eukaryotes.

The finding of p-Ser/Thr specificity in both archaeal and eukaryotic PPPs, and their shared sequence feature of a distinctive form of the “2-Arginine Clamp”, indicates that the transition between the p-Ser/p-Thr and p-Tyr bacterial PPP type and the p-Ser/Thr specific “EukaryoticPPP-Like” type must be ancient. We have attempted to infer the pattern of this transition through examination of the phylogenetic trees presented in Figs. 2, 3 and Supplemental Figure S7. However, the completeness of this picture may be impaired by the possible adverse impact of incomplete taxon representation and LGT. First, there is an almost complete absence of “EukaryoticPPP-Like” sequences from DPANN Archaeota. These organisms have small genomes, with reduced metabolic repertoires suggesting they may be symbionts or parasites of other prokaryotes^7^. As such, extensive gene lineage extinction may have removed most PPP genes. In addition, there is a paucity of representation of the Euryarchaeota, which may also indicate lineage extinction of PPP genes in many groups. The combination of these two influences may have adversely affected the tree structure at the base of the Archaea. In addition, the pattern in tree region “C” of clustering of “EukaryoticPPP-Like” sequences from the halophilic archaeal taxon MSBL-1^20^ together with bacterial sequences from mainly the Deltaproteobacteria is strongly reminiscent of results reported by others which have been interpreted as part of massive LGT between Deltaproteobacteria and Archaea^21^. Together, these less than optimal factors may have combined to render the trees more complex, with three distinct regions (“A”, “B”, “C”). Nevertheless, in both regions “A” and “B” there is a discernible pattern of clustering with Euryarchaeota sequences more basal, and Proteoarchaeota sequences more distal, suggesting a good deal of vertical inheritance of PPP genes, consistent with the structure of rooted organismal phylogenetic trees of the Archaea.

The presence of a version of the “2-Arginine Clamp” in both bacterial and archaeal PPPs begs the question of the evolutionary relationship between them. In our previous report^4^ we have traced a plausible multi-step evolutionary lineage between ancestral metallophosphoesterases (MPEs) and bacterial PPPs, which begins with nuclease-like ancestors and ends with a p-Ser/p-Thr and p-Tyr PPP like the phage lambda phosphatase (PDB:1G5B). The clamp involves key Arg residues in Motif 2 (Arg^53^ in 1G5B) and in Domain 3 (Arg^162^ in 1G5B), between Motifs 4 and 5 of lambda phosphatase. It seems unlikely a priori that such a series of intermediate changes between an ancestral nuclease-like MPE and a PPP would occur more than once. The clamp in “EukaryoticPPP-Like” sequences^4^ involves the same conserved Arg residue in Motif 2 (Arg^96^ in human PP1α) together with a second conserved Arg (Arg^221^ in human PP1α) in a specific loop. A conserved Asp (Asp^208^ in human PP1α), which has a stabilization role, has replaced the second Arg from the bacterial PPP clamp. Again, it seems unlikely that the transformation from a bacterial PPP type to a eukaryotic PPP type would have occurred more than once. This would suggest that this transition occurred in Bacteria, followed most simply, by a single colonization of Archaea by a “EukaryoticPPP-Like” ancestor sequence. A more complex scenario would envision more than one colonization by a very similar progenitor sequence type. In region “C” of our phylogenetic trees involving “EukaryoticPPP-Like” sequences from Bacteria and Archaea there is very likely evidence of LGT from the former to the latter. This is supplemented by the observation that there are variant sequences in these clusters which have characteristics which might make them intermediates in the transformation of a bacterial PPP to a “EukaryoticPPP-Like” sequence. The extreme phylogenetic restriction of “EukaryoticPPP-Like” sequences to a relatively small number of bacterial groups, chiefly Deltaproteobacteria and Firmicutes, argues that either this transformation was restricted to these groups, or at least that it only really took hold in these groups, with presumably some functional role allowing persistence of such sequence variants to the present.

The structural transformation of a nuclease-like MPE ancestral sequence type to a p-Ser/p-Thr and p-Tyr bacterial PPP, and then to a p-Ser/Thr specific “EukaryoticPPP-Like” sequence type involves remodeling of the carboxy terminus of the protein (i.e. after Motif 4). In our previous report^4^ and this study, we have presented data which documents changes to Motif 5 in the transition from ancestral MPE to bacterial PPP, and between Motif 4 and Motif 5 in the establishment of the “2-Arginine Clamp” in bacterial PPPs, and its conversion to a functionally equivalent but structurally distinct form in “EukaryoticPPP-Like” sequences. Our limited data suggests that Motif 6 may house some of the last structural changes in the establishment of truly eukaryotic PPPs. Detailed analysis of this motif shows a pattern of similarity which mirrors the structure of our phylogenetic trees, including the unexpected sister group relationship between the Methanosarcinaceae (Euryarchaeota) and eukaryotic PPPs. The conserved Tyr residue in all archaeal and eukaryotic sequences at the end of the SAPNY string (Tyr^272^ in human PP1α) would appear to have a structural stabilization role. The Cys after this Tyr residue (Cys^273^ in human PP1α) would appear to be one of the last motif features to have evolved, marking a distinction between the sequences of the Eukaryotes and those of the Methanosarcinaceae.

The early evolutionary history of eukaryotic PPPs has remained unresolved, due partly to inadequacies in the older phylogenetic tree inference methods, and also the incomplete taxon representation within various PPP classes. It has been suggested previously based upon conserved sequence signature analysis that eukaryotic PPPs probably arose as two gene groups, one encompassing PP1, Bsu1 (then called PPKL [“phosphoprotein phosphatases with kelch-like repeats”]), PP2A and PP2B; the other comprising PP5, PPEF/RdgC and PP7^22^. Our rooted Bayesian tree (Supplemental Figure S11) has confirmed this suggestion and placed it on a much firmer basis. Furthermore, we have extended these previous observations by the discovery of the group of “PP7-Like” sequences in the genus *Entamoeba*. These clearly comprise the most divergent group of eukaryotic PPP sequences yet reported. They are the most basal branch in the tree of Supplemental Figure S11.

Our data have also confirmed the previous observations that PP1, the PP2A-PP4-PP6 family, and PP5 appear to have a universal eukaryotic distribution. In contrast to earlier results, however, we have shown that Bsu1, PP7, and PPEF/RdgC sequences are more widespread than previously supposed. This is consistent with a model where both PPP gene groups arose very early in the evolution of Eukaryotes (perhaps at their inception), and then gave rise to widespread radiations, which may have been constricted somewhat secondarily with time (i.e. secondary gene losses). Our data also show that there has been more flexibility with regard to the fusion of the phosphatase domain and accessory domains than previously supposed. We presented evidence that the Bsu1 phosphatase domain became associated with two distinct accessory domains, one containing the classic Kelch repeats, the other containing EF Hands. The phylogenetic distribution of organisms with these distinct domain arrangements suggests that this divergence occurred very early in eukaryotic evolution.

The Asgard archaeota are a recently described superphylum (Lokiarchaeota, Thorarchaeota, Odinarchaeota, Heimdallarchaeota) which have the closest relationship to Eukaryotes of any living organisms, as assessed by comparisons of panels of ribosomal protein genes^23,24^. Hence an ancestral Asgard-like archaeaon is the current best candidate for a eukaryotic ancestor. In our phylogenetic trees of Fig. 2 and Supplemental Figure S7, the Asgard sequences in two clusters lie very close to the eukaryotic PPPs, with high support, consistent with these literature reports. However, unexpectedly, the closest archaeal sequence group to the eukaryotic PPPs consists of a set of sequences from organisms of the Methanosarcinaceae (Euryarchaeota). This sister-group relationship, also with high support, strongly suggests that an Asgard-like archaeal ancestor passed PPP genes to both this euryarchaeal group and the future Eukaryotes.

Lateral transfer of genes between disparate taxonomic groups might be expected to be facilitated if ancestor organisms dwelt together in close association under common environmental conditions. Many current Asgard organisms are strict anaerobes^25^, as are current methanogens^26^. This suggests that it is plausible that their ancestors might have lived in a similar common environment. A recent report^27^ has described the new Asgard species Candidatus *Prometheoarchaeum syntrophicum* MK-D1 (of the Lokiarchaeota) which was isolated and cultured from anaerobic marine sediments. This organism forms an obligate syntrophic relationship with the methanogenic archaeon *Methanogenium*. Prometheoarchaeum also forms long straight and branching cellular extensions. A model presented in this study postulates that such extensions in an ancestral archaeon might have been important in establishing intimate physical interractions with neighboring organisms—one example being the bacterial ancestor of the mitochondrion. It is certainly plausible that such an association could facilitate sharing of genetic material as well. Our phylogenetic tree data, in combination with these recent observations from a novel cultured Asgard organism, strongly suggest that such an association occurred in the past between an ancestral Asgard organism and a methanogenic ancestor, leading to the transfer of PPP genes. The PPP genes of Eukaryotes are also the apparent descendents of this ancestral Asgard gene complement.

## Materials and methods

### Database search and retrieval of candidate PPP sequences

To assess the presence of “BacterialPPP-Like” sequences in Archaea, a set of bacterial p-Ser/p-Thr and p-Tyr PPP sequences (i.e. sequences of proteins which have activity in vitro against substrates with both p-Ser/Thr and p-Tyr substrates) were collected and aligned as detailed in a previous publication^4^. A profile Hidden Markov Model (HMM) was constructed using the HMMER package^28^ (http://hmmer.org/), and used to search the archaeal protein sequence database of UniProt^29^ (http://www.uniprot.org/).

To assess the presence of “EukaryoticPPP-Like” sequences in Archaea and Bacteria a database was constructed of proteins from the completely sequenced genomes of 15 diverse eukaryotic species, collected from DOE JGI (Joint Genomes Institute) (https://genome.jgi.doe.gov/portal/), Phytozome (https://phytozome.jgi.doe.gov/pz/portal.html), MycoCosmD (https://genome.jgi.doe.gov/programs/fungi/index.jsf), and individual project websites cited in GOLD (Genomes Online Database) (https://gold.jgi.doe.gov/). PPP sequences (206) were collected utilizing a search with a PPP-specific HMM developed in a previous project^30^. Sequences were aligned utilizing the MAFFT server (see next section) and an HMM constructed as above. Another HMM was derived from the NCBI Conserved Domain Database (https://www.ncbi.nlm.nih.gov/cdd) “MPP_PPP_family” (cd07414 [MPP_PP1_PPKL]; cd07415 [MPP_PP2A_PP4_PP6]; cd07416 [MPP_PP2B]; cd07417 [MPP_PP5_C]; cd07418 [MPP_PP7]; cd07419 [MPP_Bsu1_C]; cd07420 [MPP_rdgC]) (61 sequences). Models were used to search the bacterial and archaeal protein databases from UniProt. Sequences which achieved scores in the search of greater than 80.8 bits (E < 3.3e-19) (bacterial search), or 76 bits (E < 2.8e-19) (archaeal search) were retained for processing. In addition, selected BLASTP^31^ (https://blast.ncbi.nlm.nih.gov/Blast.cgi) sequence searches were used to locate high similarity (E < 1e−30) candidate homologues at UniProt and the NCBI non-redundant protein databases. Reference eukaryotic PPP sets were obtained from the NCBI Conserved Domain database, for each of the seven subgroups listed above.

### Multiple sequence alignment

The MAFFT server^32^ (https://mafft.cbrc.jp/alignment/server/) was used to generate candidate multiple sequence alignments. These were produced under a variety of alignment options, but the most effective alignments were typically produced using the BLOSUM45 scoring matrix and iterative alignment options (E-INSI or L-INSI). In some instances sequences were added to pre-existing alignments using the MAFFT-Add feature. Alignments were evaluated both quantitatively [using the TCS (transitive consistency score^33^ function (default parameters) of the T-Coffee server^34^ (http://tcoffee.crg.cat/apps/tcoffee/do:core)] and qualitatively (for intact presence of previously characterized sequence motifs^18,35,36^.

### Reduced complexity multiple sequence alignment

An alignment with a large number of taxa (~ 485) was reduced in complexity by clustering with the CDHit technique^37–39^ (http://weizhongli-lab.org/cdhit_suite/cgi-bin/index.cgi?cmd=cd-hit) prior to phylogenetic tree analysis. Clustering was performed at 80% identity (i.e. redundant sequences with this level of identity or higher were removed). An exception was made for Asgard archaea sequences, which were all retained (they were few in number and preliminary analyses showed them to cluster in an important region of phylogenetic trees). The resulting alignment had 254 sequences.

### Phylogenetic tree inference

Maximum likelihood (ML) trees were inferred by the IQ-Tree package (v1.5.5)^40^ (http://www.iqtree.org/), running locally. The optimal model^41^ of sequence evolution was determined within IQTree 1.5.5 by a two-step procedure. Step 1: iqtree -s <AlignmentName>-m MF -mset LG -nt AUTO. The best model was taken as that producing the lowest Bayesian Information Criterion (BIC) score. This was generally of the form “LG + RX” (e.g. LG + R7), where LG is the amino acid substitution matrix of Le and Gascuel^42^, and RX is a number of “Free Rate” site-heterogeneity categories estimated from the data. Step 2: iqtree -s <AlignmentName> -m MF -mset LG -mrate G4,RX,R(X + 1),R(X + 2),G8,G12,G16,G20,G24,G28,G32 -nt AUTO. Here GX (e.g. G4) is the number of fixed gamma distribution site-heterogeneity rate categories. Once again, the best model was taken as that producing the lowest BIC (Bayesian Information Criterion) score. In general, this was LG + G32. However, there was only a modest improvement (i.e. < 5 BIC units) between the LG + G16 and LG + G32 score, hence LG + G20 was used to conserve computational resources.

Unrooted ML trees were then inferred within IQTree 1.5.5 by a procedure which is recommended for shorter sequence alignments containing many taxa^43^: iqtree -s < Alignment Name > -st AA -m LG + G20 -bb 1000 -wbtl -nm 2500 -alrt 10,000 -abayes -pers 0.2 -numstop 250 -nt AUTO. Here “-pers 0.2” sets the “perturbation strength” (a measure of the proportion of internal branches randomly rearranged during each tree search perturbation [default is 0.5]), “-numstop 250” sets the maximum number of iterations to run attempting to find a new best tree in each perturbation round [default is 100]. The best (i.e. lowest log-likelihood) tree was taken from a run of 10 replicates using identical parameters. Branch support was determined by SH-aLRT (i.e. SH-like approximate likelihood ratio test)^44^ (“-alrt 10,000”), the aBayes test^45^ (“-abayes”), and the Ultrafast bootstrap (UFBoot)^46^ (“-bb 1000”).

An unrooted Bayesian tree for the alignment clustered with CDHit at 80% identity was inferred with PhyloBayes MPI (v1.5a)^47^ implemented at the CIPRES Science Gateway^48^ (https://www.phylo.org). The evolutionary model LG + G20 was used (- lg - dgam 20), with a single fixed mixture component (-ncat 1). Data were sampled from the output of two independent chains. Chain convergence was monitored using the “maxdiff” and “effsize” parameters. For the PhyloBayes tree presented herein, maxdiff = 0.255, effsize > 400 for all parameters.

Rooted trees were inferred with BEAST (Bayesian Evolutionary Analysis by Sampling Trees)^49^ v. 1.8.4, run at the CIPRES Science Gateway V.3.3^48^ (https://www.phylo.org). BEAUTi v. 1.8.2 was used to prepare controlling XML files locally. The evolutionary model LG + G8 was used (LG + G16 or higher is not available for parallelized BEAST runs), along with a log-normal relaxed uncorrelated clock^50^. Markov Chain Monte Carlo (MCMC) was generally run for 200–250 M cycles. Data were combined from the runs of two independent chains, with the first 10% (1000) of each tree set (10,000 total) manually excluded as burn-in previously^51^.

### Harvesting of candidate sequences for the eukaryotic PPP radiation

As a benchmark for assessing the distribution and characteristics of eukaryotic PPP subtypes, a composite protein database from 45 representative Eukaryotes was assembled from DOE JGI (Joint Genomes Institute) (https://genome.jgi.doe.gov/portal/), Phytozome (https://phytozome.jgi.doe.gov/pz/portal.html), MycoCosm (https://genome.jgi.doe.gov/programs/fungi/index.jsf), and individual project websites cited in GOLD (Genomes Online Database) (https://gold.jgi.doe.gov/). This composite database was searched with our eukaryotic PPP profile HMM described above (206 sequences from 15 diverse eukaryotic species), hits with scores greater than 100 bits (E < 6.4e−27) retrieved, and sequences subjected to Batch CD Search at NCBI^52^ (https://www.ncbi.nlm.nih.gov/Structure/bwrpsb/bwrpsb.cgi). For each species the highest scoring hit of each type with a given NCBI model (cd07414 [MPP_PP1_PPKL]; cd07415 [MPP_PP2A_PP4_PP6]; cd07416 [MPP_PP2B]; cd07417 [MPP_PP5_C]; cd07418 [MPP_PP7]; cd07419 [MPP_Bsu1_C]; cd07420 [MPP_rdgC]) was taken as the representative type for that species. The resulting set of sequences was used in batch BLASTP at NCBI to obtain high scoring “self-hits”, yielding a uniform set of reference accession numbers. A representative PP1 and PP2A_PP4_PP6 family member was found for each of the 45 species—these are given, along with their species of origin, in Supplemental Table S4.

For those eukaryotic PPP types which are not universally distributed (Bsu1, PP2B, PP7, PPEF/RdgC), we searched for previously uncharacterized eukaryotic PPP sequences using an iterative database search, retrieval, and characterization procedure. As a starting point, our eukaryotic PPP profile HMM was used to search the entire complement of eukaryotic proteins in UniProt. The accession numbers of hits with scores greater than 100 bits (E < 1.2e−25) were retrieved, and used to obtain the flat file UniProt entries. These were then parsed for their taxonomy entries using a custom Python script, and this taxonomic information was sorted by major taxonomic group. Then for a given eukaryotic PPP type, entries were chosen for further examination from taxonomic groups without a known representative of that PPP type. These were subjected to Batch CD Search at NCBI. Any hits for that type were retrieved and retained as candidate sequences. At intermediate search stages for each eukaryotic PPP type (i.e. after a fresh Batch CD Search and candidate retrieval), a fresh MAFFT, BLOSUM45 alignment (EINSI or LINSI) was made with known literature sequences of that PPP type, plus the new candidates, as described above. From this alignment a new profile HMM would be made by the HMMER package. This HMM would then be used to search the UniProt eukaryotic protein database again, and another search round initiated. In addition, to prevent the sensitivity of the NCBI models for standard eukaryotic PPP types from limiting discovery of possible novel candidate PPP sequences, during some search stages sequences were also processed which were too divergent to get hits with the above set of standard PPP models, but which did get a hit with the more generic model cd00144 (MPP_PPP_Family).

After all available hits for each eukaryotic PPP sequence type were obtained, the entire set was combined with the reference set of PP1, PP2A_PP4_PP6, and PP2B sequences obtained from the benchmark set of 45 eukaryotic species described above. The identities of all these candidates were then validated by multiple sequence alignment and maximum likelihood phylogenetic tree inference, as described above. As our benchmark 45 eukaryotic species dataset indicated that PP5s appear to be universally distributed, no concerted attempt was made to identify new PP5 sequences. A few novel sequences were obtained secondary to searching for other sequences (see below) and these were included with our results.

For candidate sequences of each eukaryotic PPP type, the presence of possible accessory domains was first assessed by reference to the Batch CD Search results described above. In a few instances more divergent domains were found by use of HHPred^53,54^ (https://toolkit.tuebingen.mpg.de/#/tools/hhpred) and HHrepID (https://toolkit.tuebingen.mpg.de/#/tools/hhrepid) at the MPI Bioinformatics Toolkit^55,56^ (https://toolkit.tuebingen.mpg.de/#/).

An exception to the above search procedure was necessary in the case of PPEF/RdgC sequences. Initial alignments were made using sequences from the literature^10^, and the resulting HMMs used to search eukaryotic proteins at UniProt. However, during screening of these candidates, experience showed that the NCBI model (cd07420 [MPP_rdgC]) was only capable of retrieving sequences of metazoan origin. However, known PPEF/RdgC sequences from the literature achieved cross-hits with the NCBI PP5 model (cd07417 [MPP_PP5_C]), though with reduced scores (< 100 bits). Thereafter, sequences which obtained weak hits (< 100 bits) with this model which also lacked accompanying TPR domain hits were processed as possible PPEF/RdgC sequences. The same stepwise procedure was then followed as above, where intermediate alignments and HMMs were made during the stages of the search process.

### WebLogo analysis and corresponding sequence alignments

The WebLogo3 server (http://weblogo.threeplusone.com/create.cgi)^51,57^ was used to generate graphical representations of sequence conservation in Motif 6. Server settings were left at their defaults with the exception of the following: Sequence type (Protein); Logo-size (large); Color scheme (Chemistry (AA)). The starting sequence alignment for this analysis was what is presented as Supplemental Figure S5. Eukaryotic PPP and “EukaryoticPPP-Like” sequences were retained, while bacterial PPP and “BacterialPPP-Like” sequences were dropped. The detailed phylogenetic tree of Supplemental Figure S7 was used to access species names of sequences in various tree regions. These were then used together with Supplemental Tables S2 and S3 to access primary sequence numbers. These were then used with the large parent alignment to pull out and compose the subsets needed: eukaryotic PPP; archaeal PPP—Methanosarcinaceae; archaeal PPP—tree region “B”; archaeal PPP—tree region “A”; archaeal and bacterial PPP—tree region “C”. Individual sub-alignments were then edited to the Motif 6 region. The subalignments corresponding to each WebLogo3 representation were then collected and presented as Supplemental Figure S9.

## Supplementary Information

Supplementary Figures.

Supplementary Tables.
