# Supplementary material for: The origin and radiation of the phosphoprotein phosphatase (PPP) enzymes of Eukaryotes
Source: Sci Rep. 2021 Jul 1;11:13681. doi: 10.1038/s41598-021-93206-8 (PMC8249667; doi:10.1038/s41598-021-93206-8)
Supplement: Supplementary file 1 — Supplementary Figures. [file 41598_2021_93206_MOESM1_ESM.pdf]

The origin and radiation of the phosphoprotein phosphatase (PPP) enzymes of Eukaryotes

David Kerk, Jordan F. Mattice, Mario E. Valdés-Tresanco, Sergei Yu Noskov, Kenneth K.-S. Ng, & Greg B. Moorhead

Supplementary Information

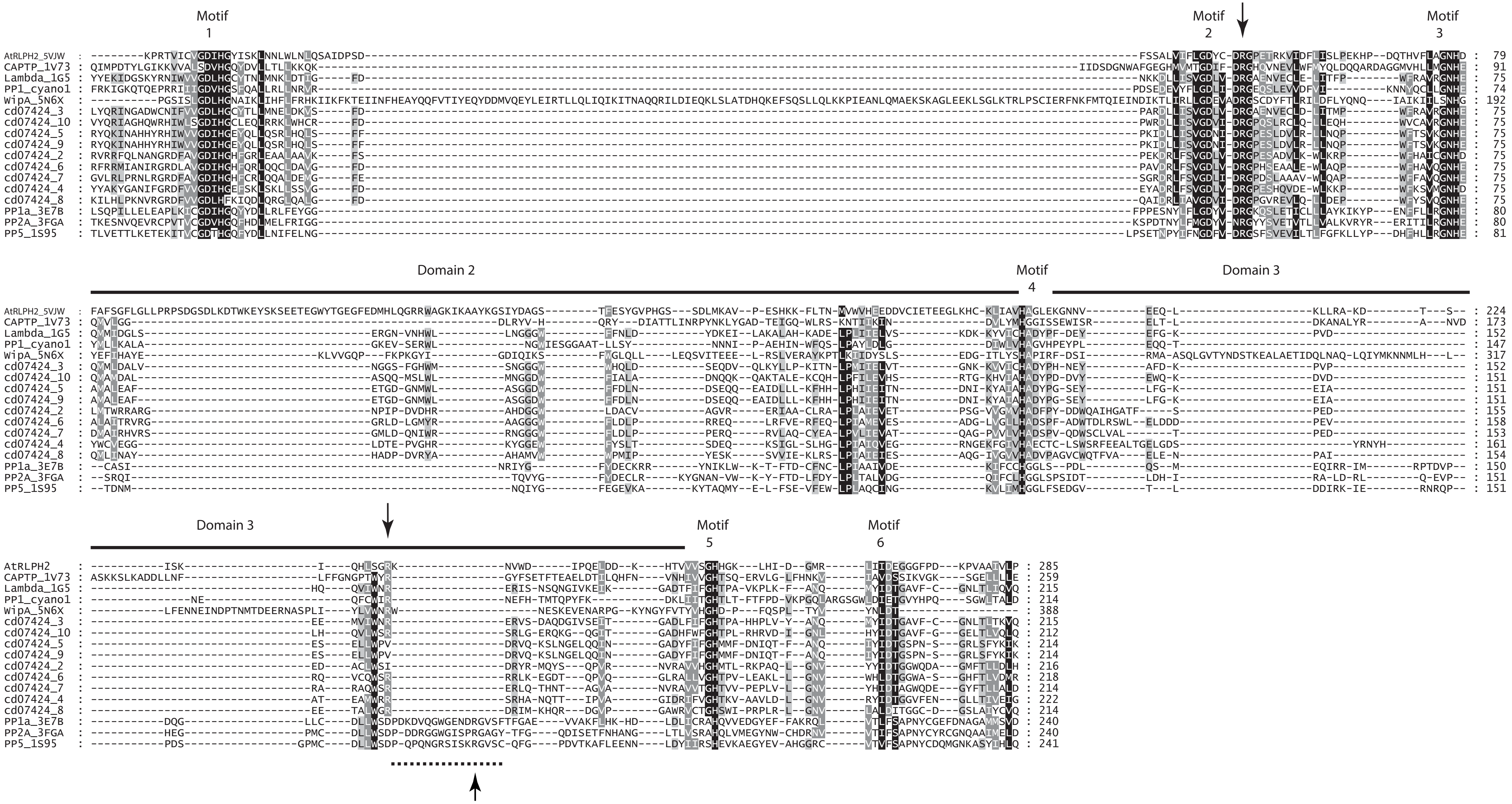

Supplemental Figure S1: Structure-guided alignment of bacterial and eukaryotic PPPs.

The sequence PP1-cyano1 was added to the structural alignment of Supplemental Figure S12 in 1, using MAFFT-Add (BLOSUM45, LINSI). Then cd07424s were added to this alignment using MAFFT-Add (BLOSUM45, LINSI). Conserved sequence motifs are labeled. Domains 2 and 3 are labeled. The downward arrow in Motif 2 denotes the Arg residue which is conserved in the first position of the "2-Arginine Clamp" 1 of both bacterial PPP and eukaryotic PPP sequences. The downward arrow in Domain 3 denotes the Arg residue which is conserved in the second position of the "2-Arginine Clamp" of bacterial PPPs. The dotted underline in Domain 3 indicates a sequence loop which is restricted to eukaryotic PPPs. The upward arrow in Domain 3 denotes the Arg residue which is conserved in the second position of the "2-Arginine Clamp" in eukaryotic PPPs. The sequences at the top and the bottom of the alignment are identified by their 4-letter PDB codes. "PP1-cyano1" and the cd07424 set of sequences are listed in Supplemental Table S5 along with their species of origin and their database accession numbers.

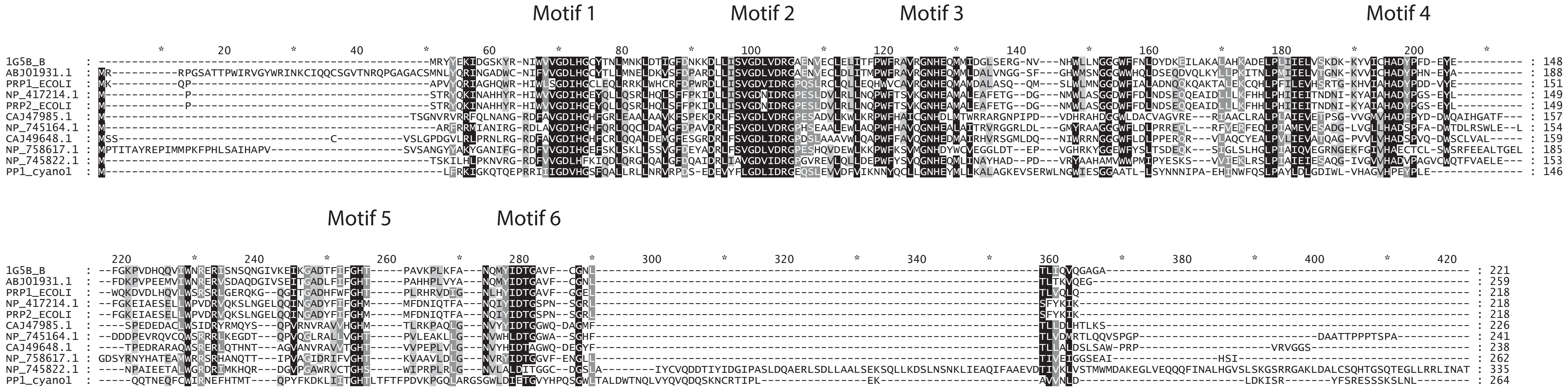

Supplemental Figure S2: Alignment underlying p-Ser/p-Thr and p-Tyr bacterial PPP HMM

p-Ser/p-Thr and p-Tyr bacterial PPP sequences (i.e. those with activity against p-Ser/Thr and p-Tyr substrates in vitro) were collected and aligned as detailed in a previous report 1. A profile HMM was then constructed as detailed in Methods. Sequences are identified either by their 4-letter PDB codes, NCBI or UniProt database accession numbers. The UniProt accession number and species of origin for "PP1-cyano1" is given in Supplemental Table S5.

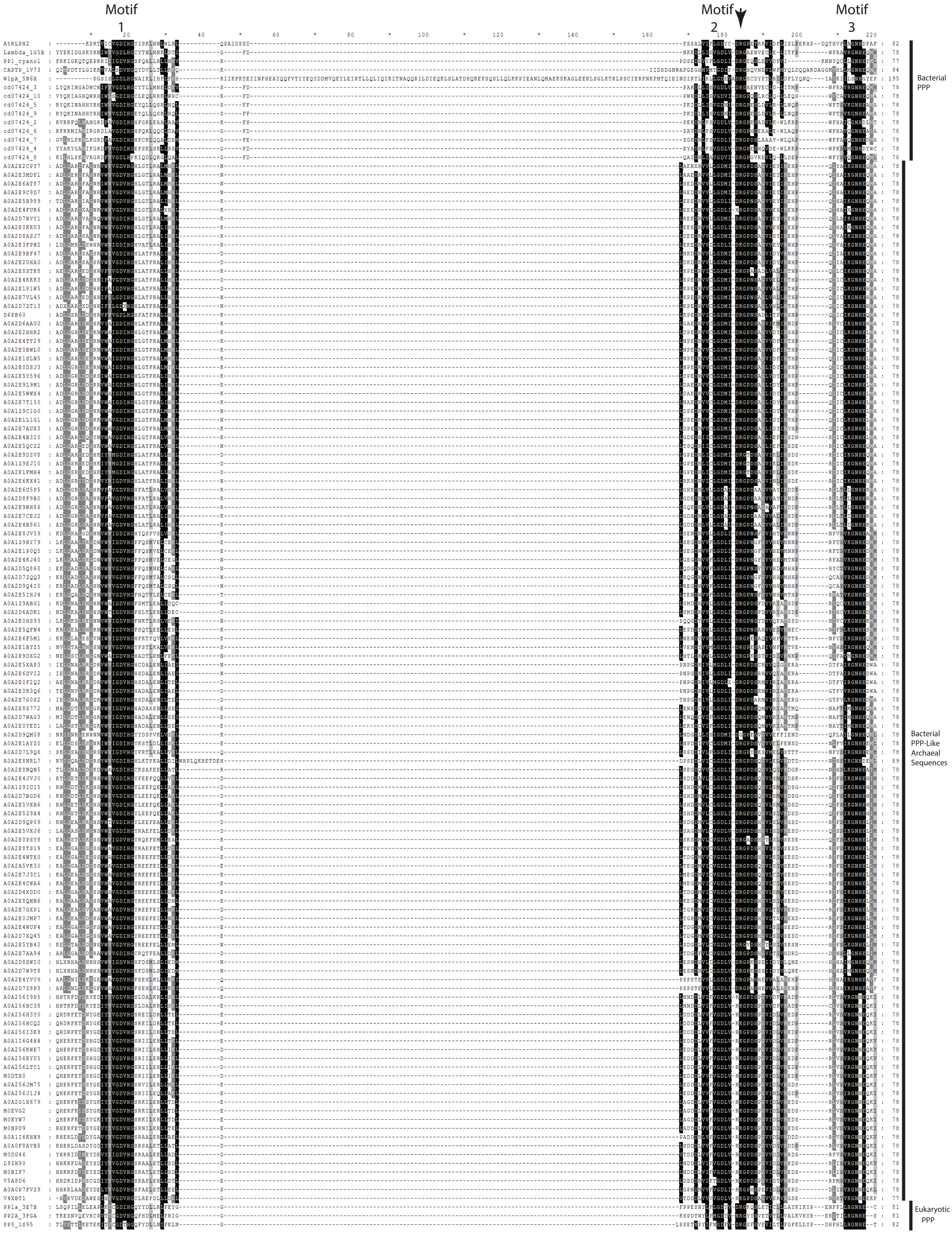
Motif 1
Motif 2
Motif 3
Bacterial PPP
Bacterial PPP-Like Archaeal Sequences
Eukaryotic PPP

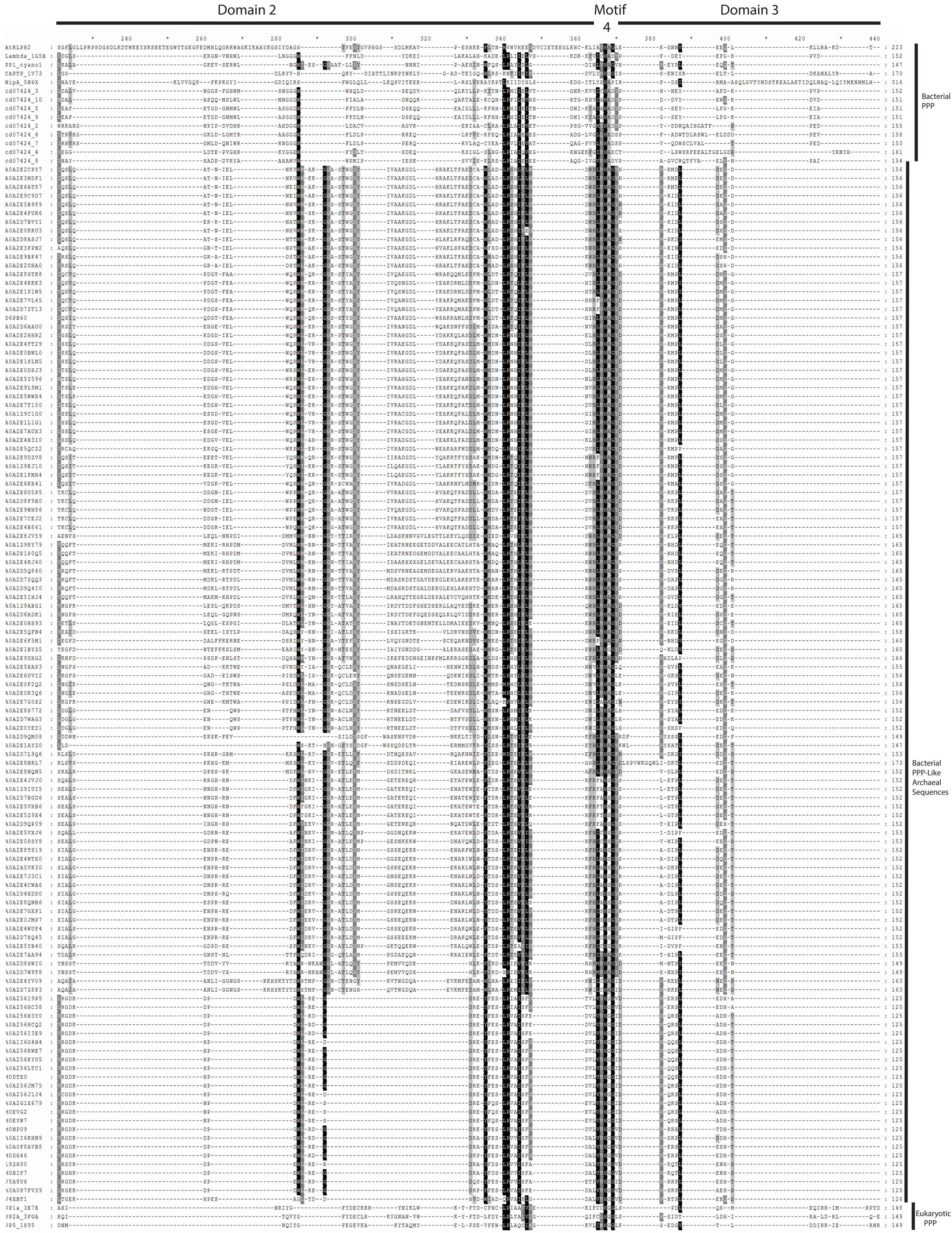

Domain 2
Motif 4
Domain 3
Bacterial PPP
Bacterial PPP-Like Archaeal Sequences
Eukaryotic PPP

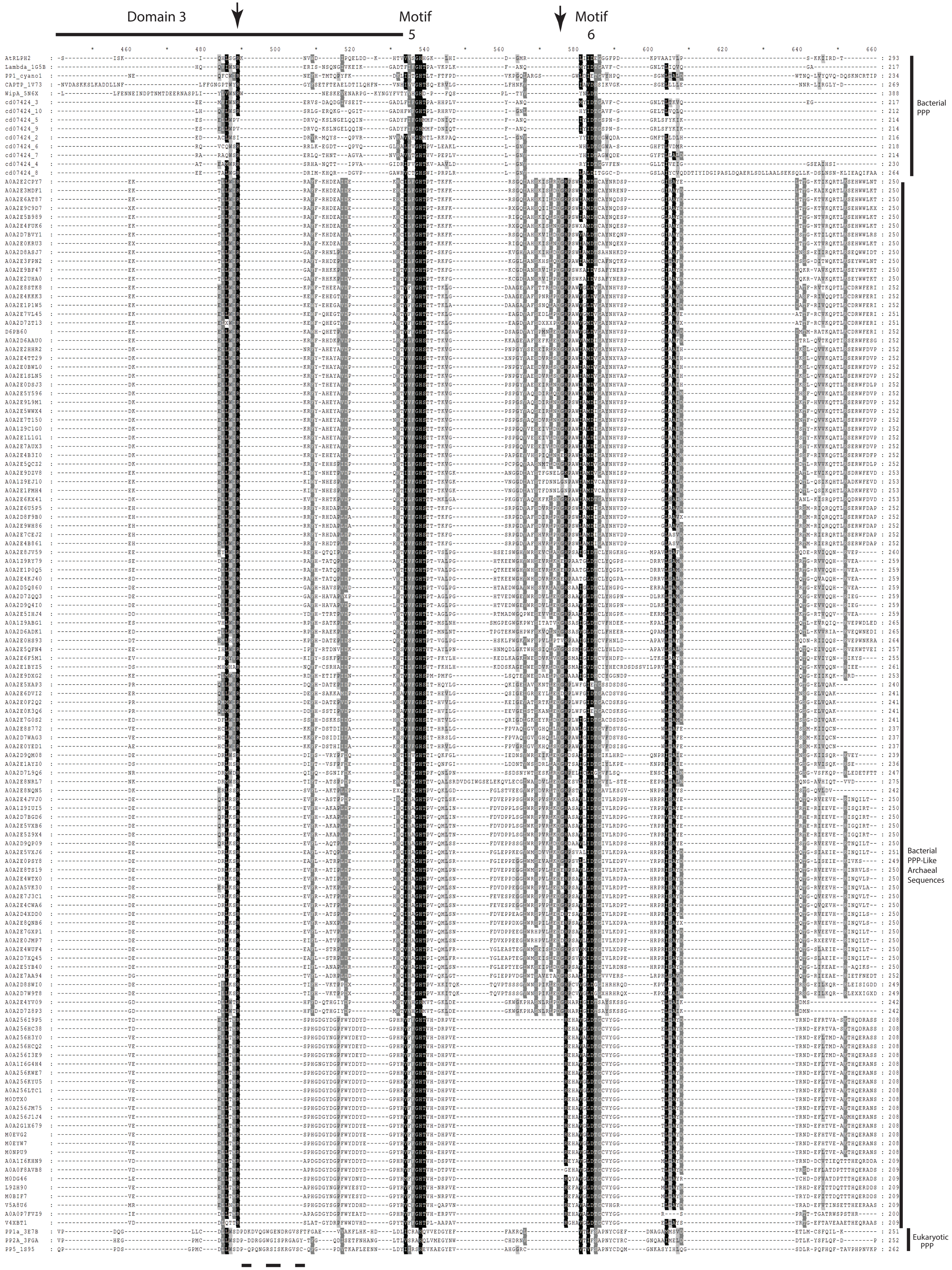
Domain 3
Motif 5
Motif 6
Bacterial PPP
Bacterial PPP-Like Archaeal Sequences
Eukaryotic PPP

Supplemental Figure S3: Structure-guided alignment of bacterial PPPs, archaeal "BacterialPPP-Like" candidate sequences, and eukaryotic PPPs

To the structure-guided alignment of Supplemental Figure S1 were added "BacterialPPP-Like" sequences from Archaea, obtained through HMM search, using MAFFT-Add (BLOSUM45, LINSI). The archaeal candidate sequences are presented in Supplemental Table S1. Conserved sequence motifs are labeled. Domains 2 and 3 are labeled. The downward arrows in Motif 2 and Domain 3 denote the structurally conserved Arg residues which constitute the "2-Arginine Clamp" in the bacterial PPPs and the archaeal "BacterialPPP-Like" sequences. The downward arrow preceding Motif 6 denotes an additional Arg sequence position which is conserved in the archaeal "BacterialPPP-Like" sequences. The dashed underline in Domain 3 denotes the sequence loop which is specific for eukaryotic PPPs. The upward arrow beneath this underline denotes the second Arg residue of the "2-Arginine Clamp" of eukaryotic PPPs. . "AtRLPH2" is the solved structure 5VJW. The database accession numbers and species of origin for "PP1-cyano1" and the cd07424 sequences are given in Supplemental Table S5. The names of the four sequences at the bottom of the alignment represent 4-letter PDB codes. All other sequence names are UniProt accession numbers, which are listed in Supplemental Table S1.

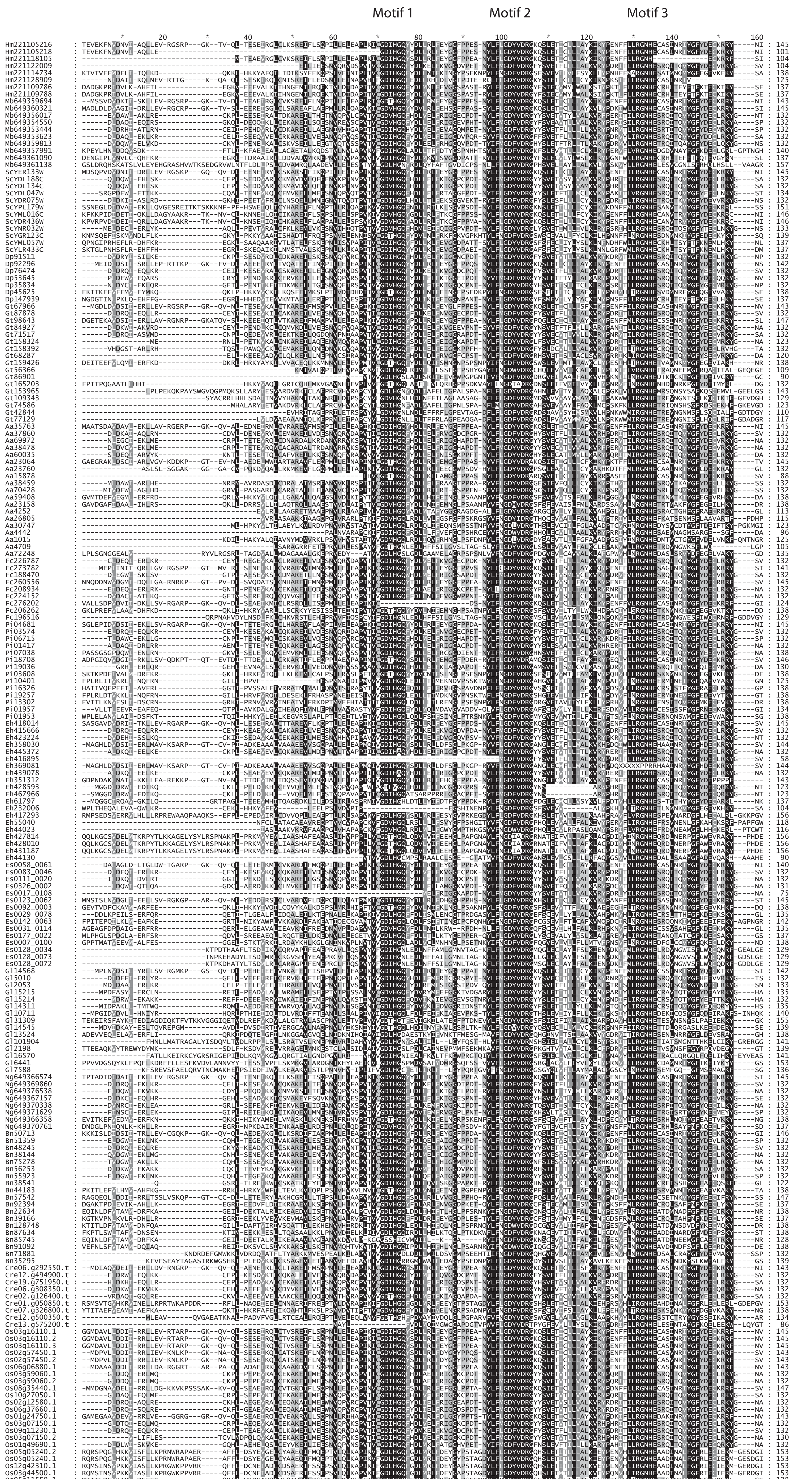
Motif 1
Motif 2
Motif 3

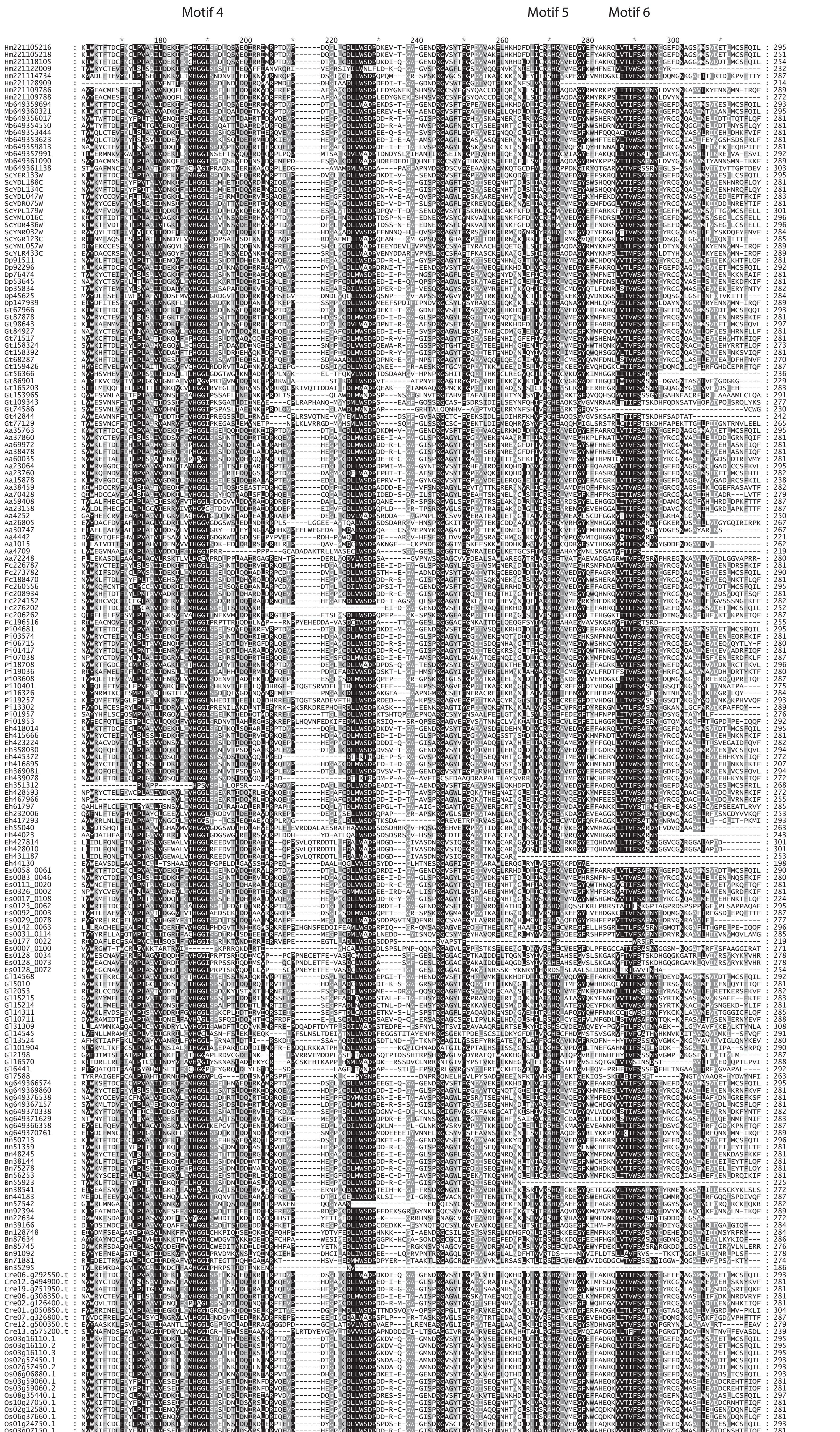

Supplemental Figure S4: Alignment underlying eukaryotic PPP HMM

PPP sequences from reference eukaryotic genomes were collected as detailed in Methods. Sequences were aligned at the MAFFT server, and a profile HMM constructed as detailed in Methods. This alignment contains PPP sequences from 15 species, which are designated in the alignment as follows: Hm (Hydra magnipapillata); Mb (Monosiga brevicollis); Sc (Saccharomyces cerevisiae); Dp (Dictyostelium purpureum); Gt (Guillardia theta); Aa (Aureococcus anophagefferens); Fc (Fragilariopsis cylindrus); Pi (Phytophthora infestans); Eh (Emiliania huxleyi); Es (Ectocarpus siliculosus); Gl (Giardia lamblia); Ng (Naegleria gruberi); Bn (Bigelowiella natans); Cre (Chlamydomonas reinhardtii); Os (Oryza sativa). The sequence alignment names and NCBI/JGI database accession numbers are listed in Supplemental Table S5.

Supplemental Figure S5

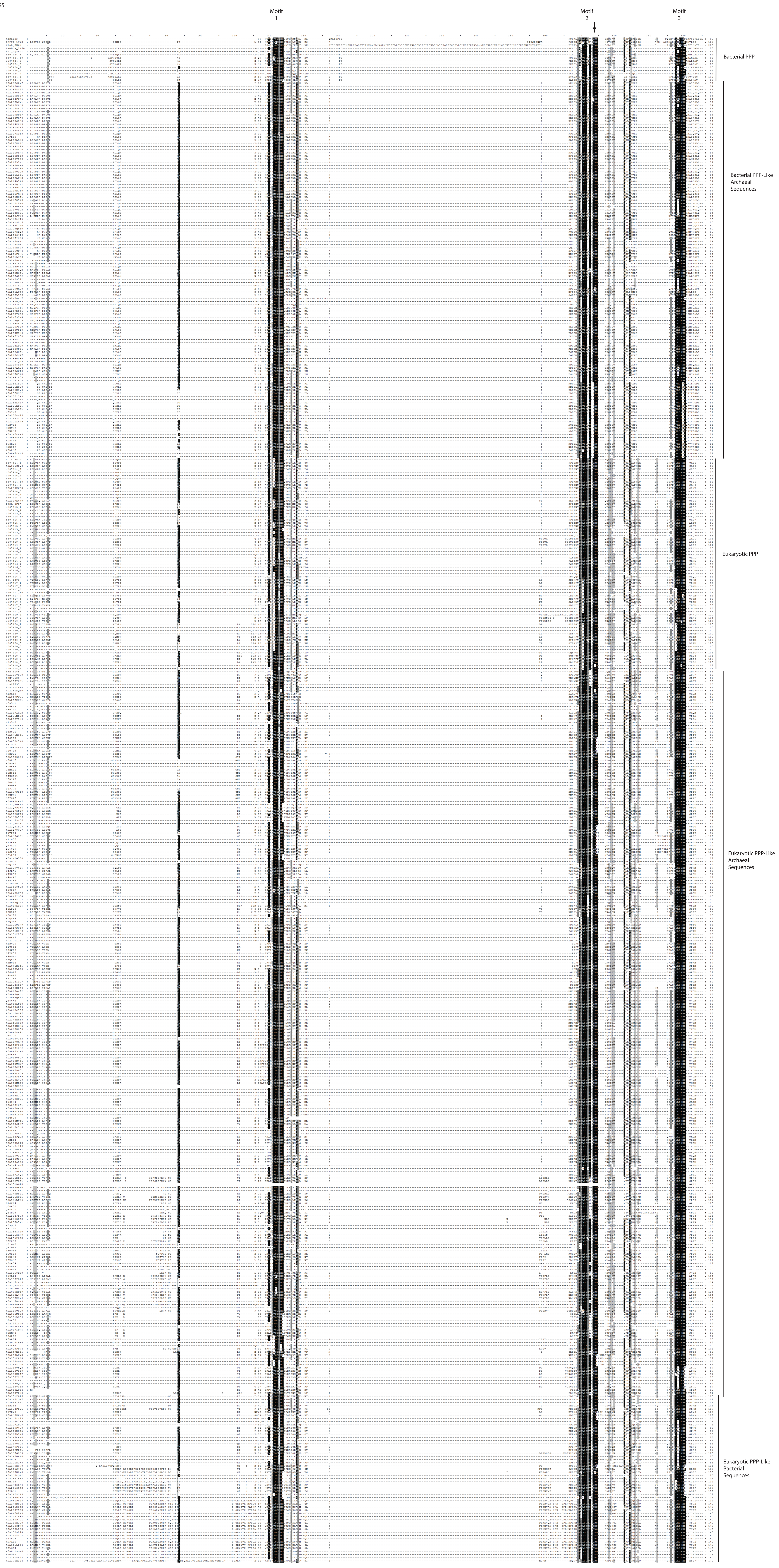

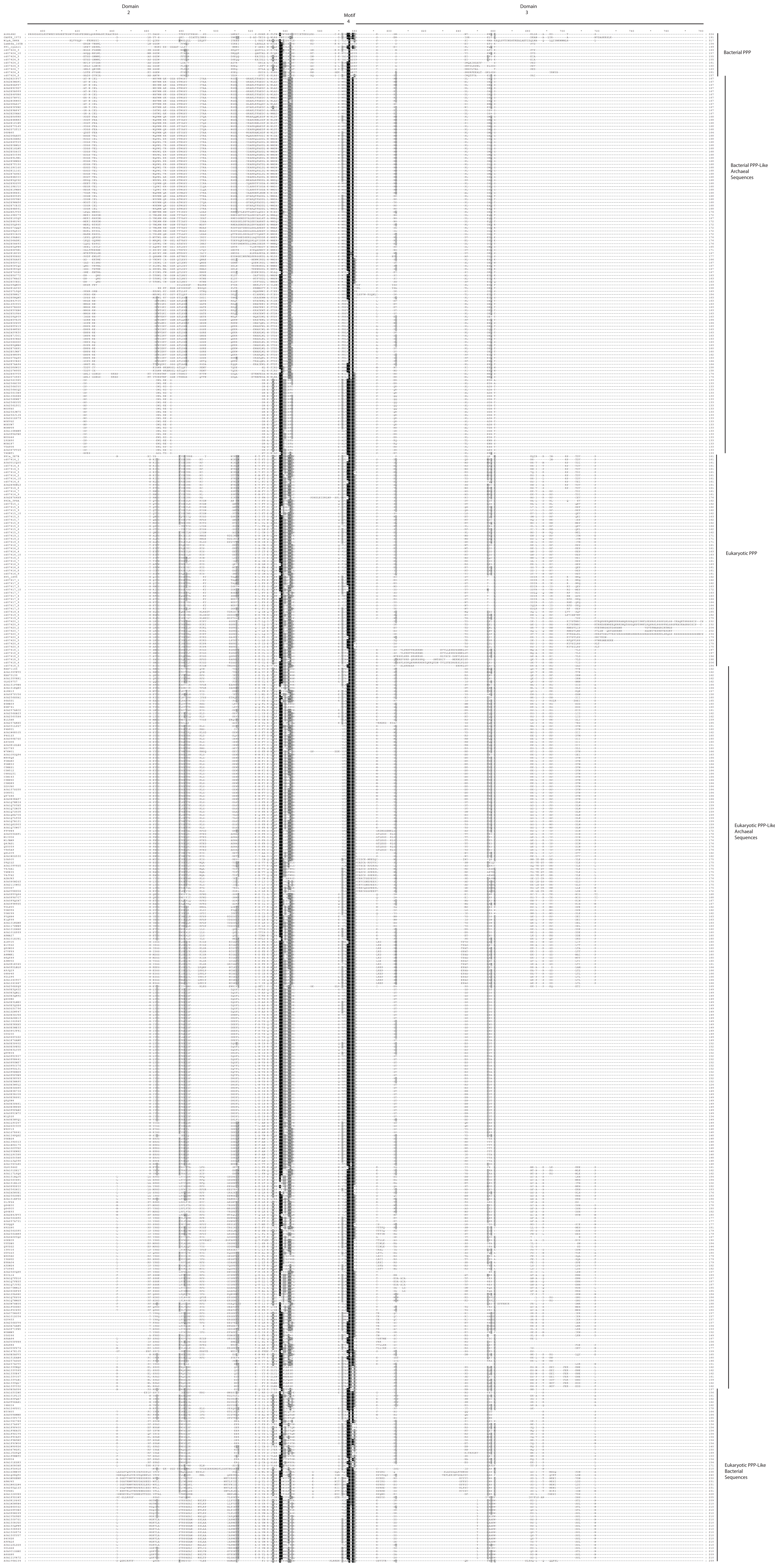

Domain 2
Motif 4
Domain 3
Bacterial PPP
Bacterial PPP-Like Archaeal Sequences
Eukaryotic PPP
Eukaryotic PPP-Like Archaeal Sequences
Eukaryotic PPP-Like Bacterial Sequences

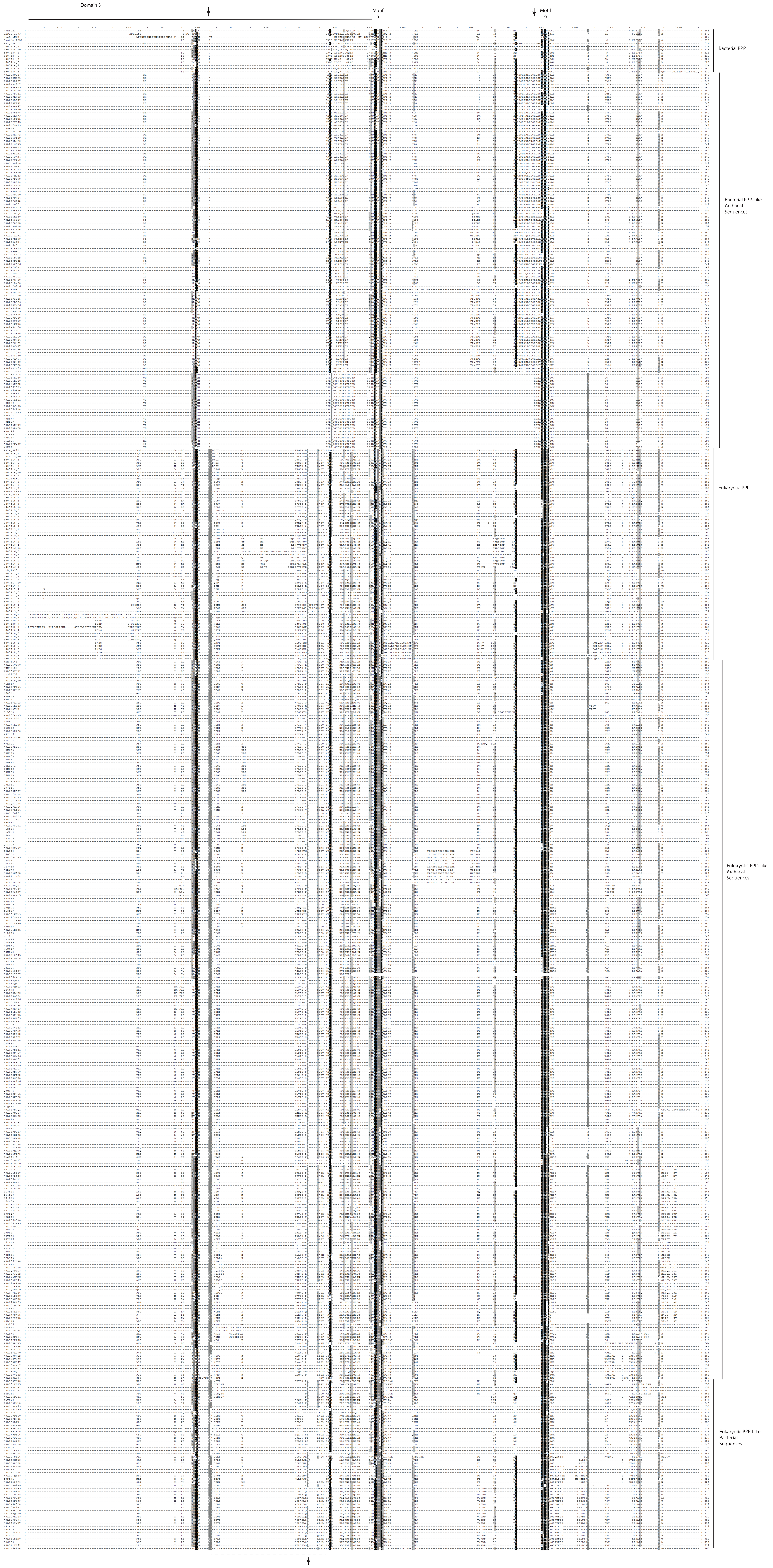

Domain 3
Motif 5
Motif 6
Bacterial PPP
Bacterial PPP-Like Archaeal Sequences
Eukaryotic PPP
Eukaryotic PPP-Like Archaeal Sequences
Eukaryotic PPP-Like Bacterial Sequences

Supplemental Figure S5: Structure-guided alignment of bacterial PPPs, eukaryotic PPPs, archaeal "BacterialPPP-Like" candidate sequences, archaeal "EukaryoticPPP-Like" candidate sequences, and bacterial "EukaryoticPPP-Like" candidate sequences

To the structure-guided alignment of Supplemental Figure S3 were added the NCBI CD set of eukaryotic PPPs, the archaeal "EukaryoticPPP-Like" sequences obtained by HMM search, and the bacterial "EukaryoticPPP-Like" sequences obtained by HMM search, using MAFFT-Add (BLOSUM45, LINSI). Conserved motifs are labeled. Domains 2 and 3 are labeled. The downward arrow in Motif 2 denotes the Arg residue which constitutes the first part of the "2-Arginine Clamp" in both bacterial and eukaryotic PPP sequences. The downward arrow in Domain 3 denotes the second Arg residue which completes the clamp in the bacterial PPPs and the archaeal "BacterialPPP-Like" sequences. These Arg residues are replaced by Asp residues in eukaryotic PPPs and "EukaryoticPPP-Like" sequences from Archaea and Bacteria. The downward arrow preceding Motif 6 denotes an additional Arg sequence position which is conserved in the archaeal "BacterialPPP-Like" sequences. The dashed line in Domain 3 denotes a sequence loop which is found in eukaryotic PPPs, and "EukaryoticPPP-Like" sequences from Archaea and Bacteria. The upward arrow within this loop denotes the second Arg residue which completes the "2-Arginine Clamp" in eukaryotic PPPs, and is also conserved in most of the archaeal and bacterial "EukaryoticPPP-Like" sequences. Three bacterial sequences are found within the eukaryotic PPP group, and are apparent lateral gene transfers from Eukaryotes to Bacteria: A0A0U1CQY0 (Chlamydia trachomatis), A0A2E9XWL3 (Rickettsiales bacterium), and A0A2E7X4X9 (Rhodobacteraceae bacterium). The component candidate sequences are summarized in Supplemental Table S1 (archaeal candidates, "BacterialPPP-Like"), Supplemental Table S2 (archaeal candidates, "EukaryoticPPP-Like"), and Supplemental Table S3 (bacterial candidates, "EukaryoticPPP-Like"). "). "AtRLPH2" is the solved structure 5VJW. The database accession numbers and species of origin for "PP1-cyano1" and the cd07424 sequences are given in Supplemental Table S5. The database accession numbers and species of origin of cd07414, cd07415, cd07416, cd07417, cd07418, cd07419 and cd07420 sequences are given in Supplemental Table S5. All other sequence names are UniProt accession numbers and are listed in Supplemental Tables S1-S3.

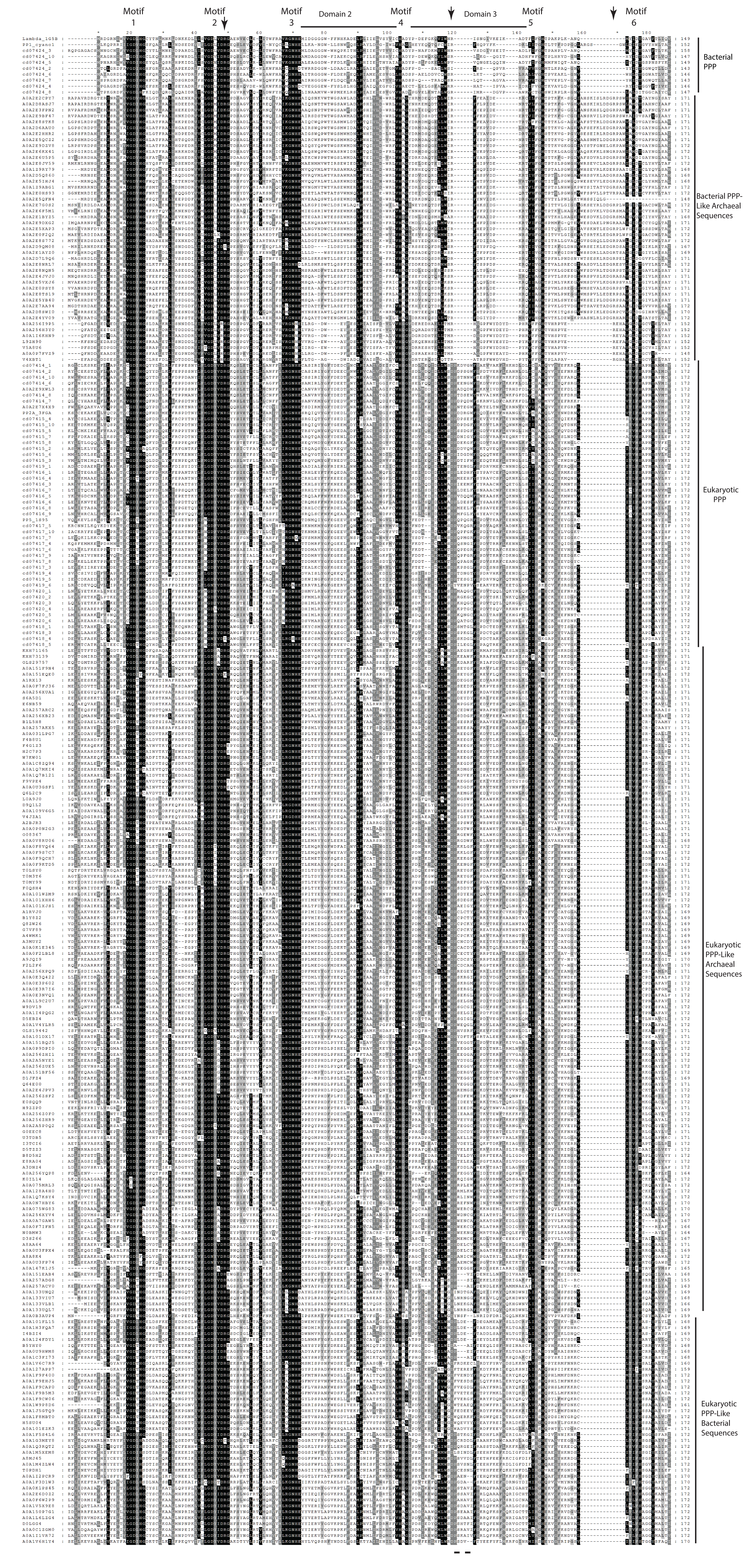
Motif 1
Motif 2
Motif 3
Domain 2
Motif 4
Domain 3
Motif 5
Motif 6
Bacterial PPP
Bacterial PPP-Like Archaeal Sequences
Eukaryotic PPP
Eukaryotic PPP-Like Archaeal Sequences
Eukaryotic PPP-Like Bacterial Sequences

Supplemental Figure S6: Edited alignment of PPP sequences from Archaea, Bacteria, and Eukaryotes

This is an edited version of the structure-guided sequence alignment presented as Supplemental Figure S5. It includes bacterial PPPs, eukaryotic PPPs, archaeal "BacterialPPP-Like" candidate sequences, archaeal "EukaryoticPPP-Like" candidate sequences, and bacterial "EukaryoticPPP-Like" candidate sequences. Conserved motifs are labeled. Domains 2 and 3 are labeled. The downward arrow in Motif 2 denotes the Arg residue which constitutes the first part of the "2-Arginine Clamp" in both bacterial and eukaryotic PPP sequences. The downward arrow in Domain 3 denotes the second Arg residue which completes the clamp in the bacterial PPPs and the archeal "BacterialPPP-Like" sequences. These Arg residues are replaced by Asp residues in eukaryotic PPPs and "EukaryoticPPP-Like" sequences from Archaea and Bacteria. The dashed line in Domain 3 denotes a sequence loop which is found in eukaryotic PPPs, and "EukaryoticPPP-Like" sequences from Archaea and Bacteria. The arrow preceding Motif 6 denotes an additional Arg sequence position which is conserved in the archaeal "BacterialPPP-Like" sequences. Three bacterial sequences are found within the eukaryotic PPP group, and are apparent lateral gene transfers from Eukaryotes to Bacteria: A0A0U1CQY0 (Chlamydia trachomatis), A0A2E9XWL3 (Rickettsiales bacterium), and A0A2E7X4X9 (Rhodobacteraceae bacterium). The TCS score (see Methods) of this alignment is 870. The database accession numbers and species of origin for "PP1-cyano1" and the cd07424 sequences are given in Supplemental Table S5. The database accession numbers and species of origin of cd07414, cd07415, cd07416, cd07417, cd07418, cd07419 and cd07420 sequences are given in Supplemental Table S5. All other sequence names are UniProt accession numbers and are listed in Supplemental Tables S1-S3.

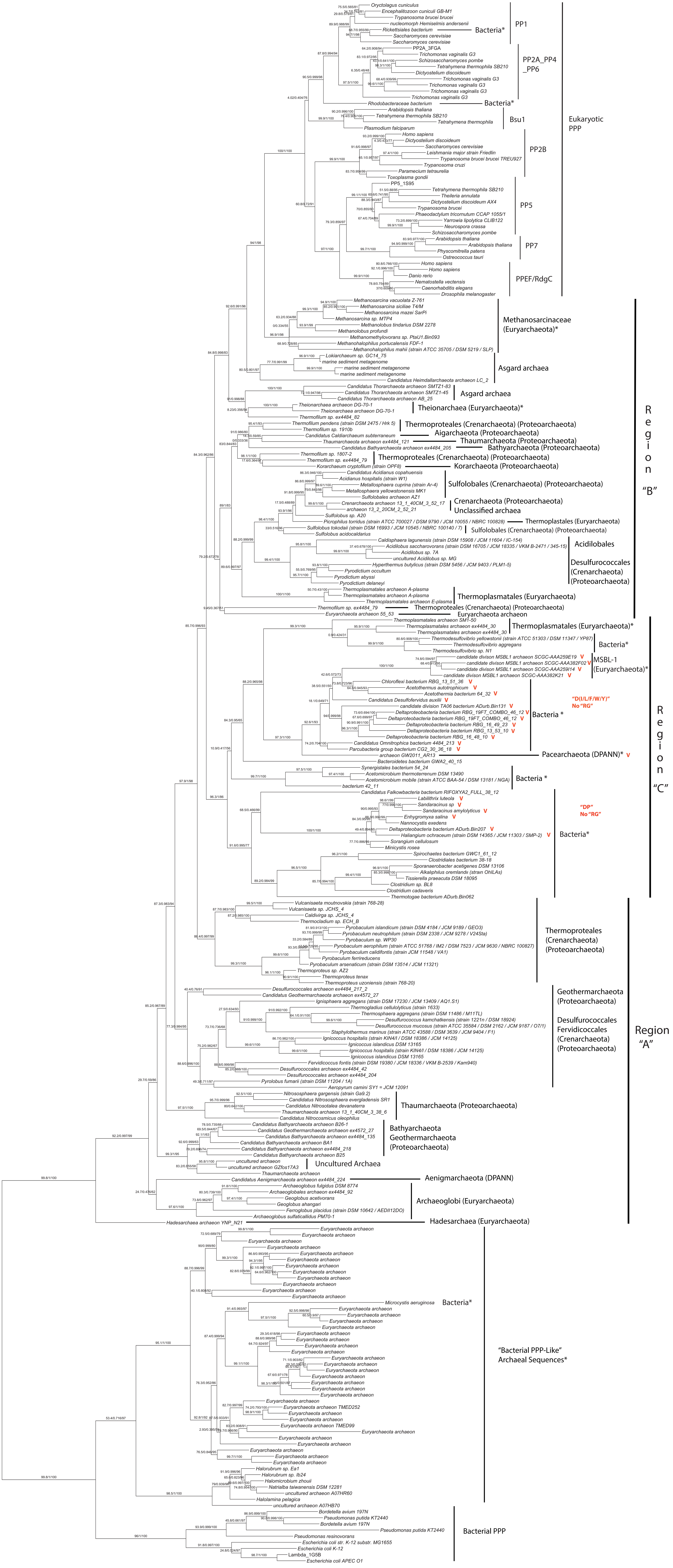

Bacteria*
PP1
PP2A_PP4 _PP6
Bacteria*
Bsu1
Eukaryotic PPP
PP2B
PP5
PP7
PPEF/RdgC
Methanosarcinaceae (Euryarchaeota)*
Asgard archaea
Asgard archaea
Theionarchaea (Euryarchaeota)*
Thermoproteales (Crenarchaeota) (Proteoarchaeota)
Aigarchaeota (Proteoarchaeota)
Thaumarchaeota (Proteoarchaeota)
Bathyarchaeota (Proteoarchaeota)
Thermoproteales (Crenarchaeota) (Proteoarchaeota)
Korarchaeota (Proteoarchaeota)
Sulfolobales (Crenarchaeota) (Proteoarchaeota)
Crenarchaeota (Proteoarchaeota) Unclassified archaea
Thermoplastales (Euryarchaeota)
Sulfolobales (Crenarchaeota) (Proteoarchaeota)
Acidilobales
Desulfurococcales (Crenarchaeota) (Proteoarchaeota)
Thermoplasmatales (Euryarchaeota)
Thermoproteales (Crenarchaeota) (Proteoarchaeota)
Euryarchaeota archaeon
Region "B"
Thermoplasmatales (Euryarchaeota)*
Bacteria*
MSBL-1 (Euryarchaeota)*
"D(I/L/F/W/Y)" No "RG"
Bacteria *
Pacearchaeota (DPANN)*
Bacteria *
"DP" No "RG"
Bacteria*
Region "C"
Thermoproteales (Crenarchaeota) (Proteoarchaeota)
Geothermarchaeota (Proteoarchaeota)
Desulfurococcales Fervidicoccales (Crenarchaeota) (Proteoarchaeota)
Thaumarchaeota (Proteoarchaeota)
Bathyarchaeota Geothermarchaeota (Proteoarchaeota)
Uncultured Archaea
Aenigmarchaeota (DPANN)
Archaeoglobi (Euryarchaeota)
Hadesarchaea (Euryarchaeota)
Region "A"
Bacteria*
"Bacterial PPP-Like" Archaeal Sequences*
Bacterial PPP

Supplemental Figure S7: Evolution of PPP sequences in Archaea, Bacteria, and Eukaryotes – detailed phylogenetic tree.

Candidate PPP sequences were collected by HMM (Hidden Markov Model) based search methods, aligned, and phylogenetic trees inferred as detailed in Methods. Shown is a detailed unrooted orthogonal phylogram. The topology depicted is from the Maximum Likelihood (ML) tree. Support numbers on each branch represent SH-aLRT/aBayes/UFBoot (see Methods for details). A simplified cartoon representation of this orthogonal phylogram is presented as Figure 2, and a radial phylogram is presented as Figure 3. Regions of the tree are labeled "A", "B" and "C" as in Figure 3 (discussed in Text). In the trees, individual sequences or groups whose placement is likely to be due to lateral gene transfer (LGT) (see Text) have an asterisk. Sequences denoted with a bold "V" symbol in orange have variant residues within the "EukaryoticPPP-Like" specific loop in Domain 3 (discussed in Text). A summary of these variations is given in bold orange text in the Figure. The alignment giving rise to these trees is presented as Supplemental Figure S6. The component candidate sequences are summarized in Supplemental Table S1 (archaeal candidates, "BacterialPPP-Like"), Supplemental Table S2 (archaeal candidates, "EukaryoticPPP-Like"), and Supplemental Table S3 (bacterial candidates, "EukaryoticPPP-Like"), and Supplemental Table S5.

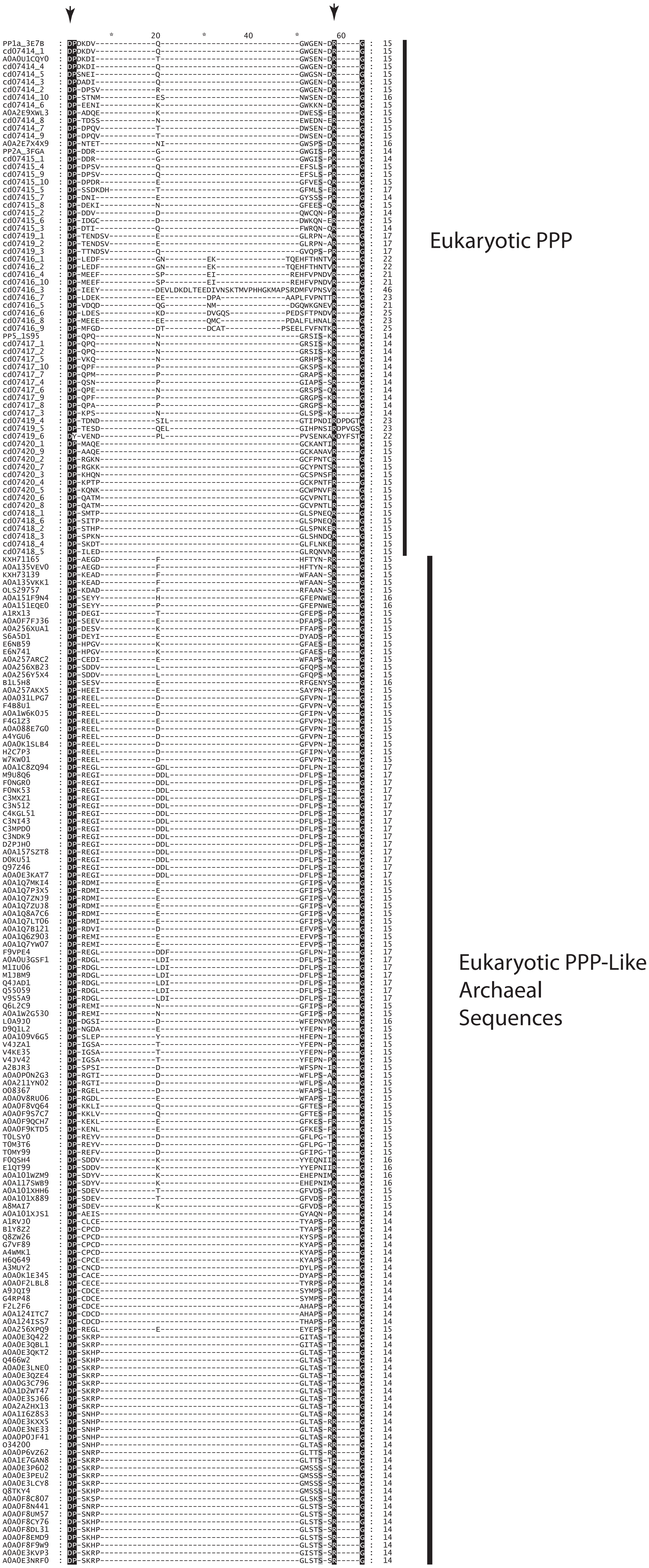

Eukaryotic PPP
Eukaryotic PPP-Like Archaeal Sequences

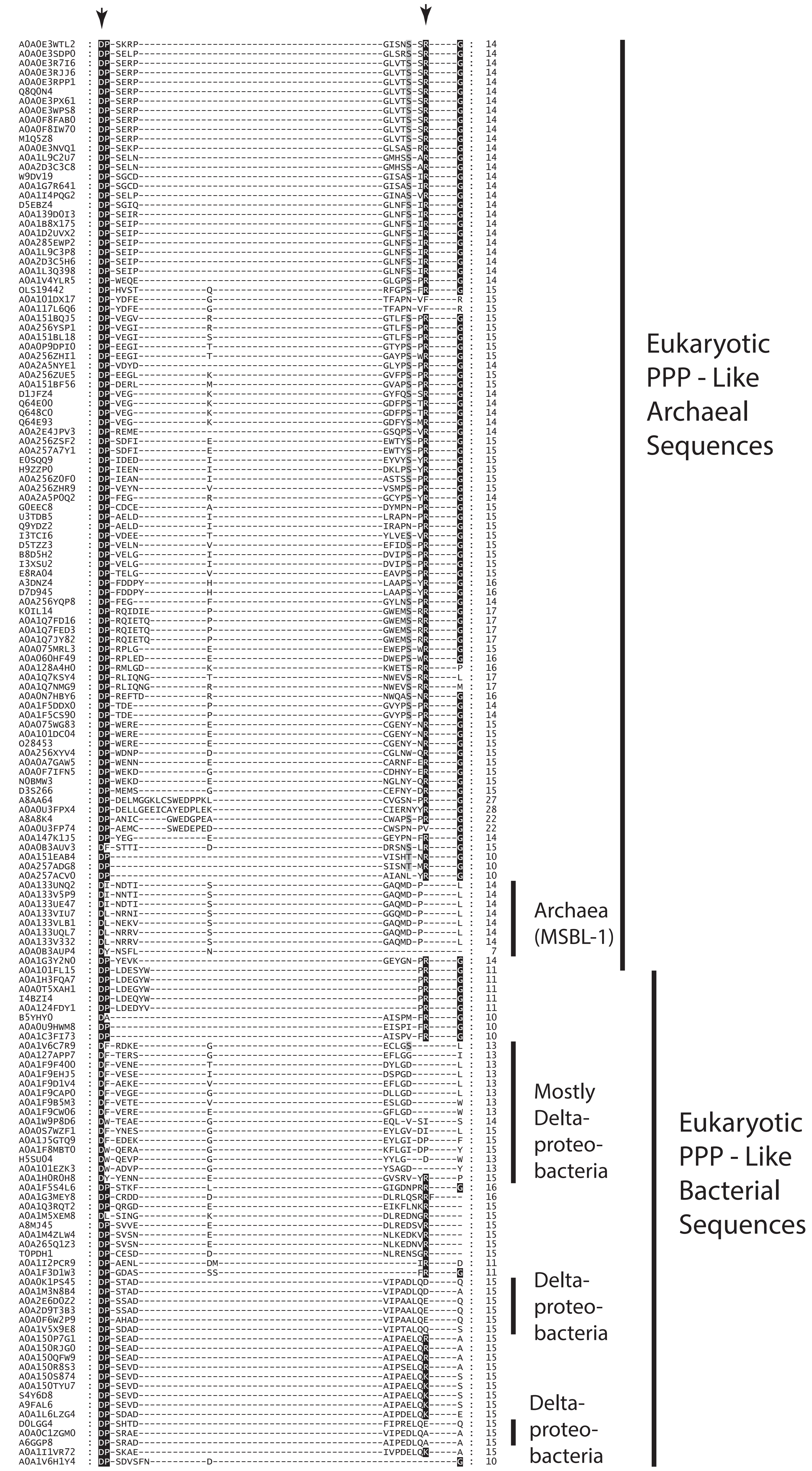

Supplemental Figure S8: Detail of a sequence loop restricted to eukaryotic PPPs and "EukaryoticPPP-Like" sequences

This figure presents an isolated view of the sequence loop restricted to eukaryotic PPP sequences and "EukaryoticPPP-Like" sequences of Archaea and Bacteria, whose complete alignment was presented in Supplemental Figure S5. The left downward arrow designates the Asp residue (Asp208 in human PP1) which is conserved in all these sequences. The right downward arrow designates the Arg residue (Arg221 in human PP1) which is the second residue of the "2-Arginine Clamp" in eukaryotic PPPs and is conserved in most of these sequences. The conservation of the "DP" and "RG" doublets is discussed in the Text. Toward the bottom of the alignment are indicated sequence groups where variations on this pattern occur. These variations are discussed in the Text and are indicated in the detailed phylogenetic tree of Supplemental Figure S7 in bold orange text letters. Sequences showing these variant patterns are indicated in Supplemental Figure S7 with a bold orange "V". Sequences from solved structures are indicated by their 4-letter PDB codes. The database accession numbers and species of origin of cd07414, cd07415, cd07416, cd07417, cd07418, cd07419 and cd07420 sequences are given in Supplemental Table S5. All other sequence names are UniProt accession numbers and are listed in Supplemental Tables S2 and S3.

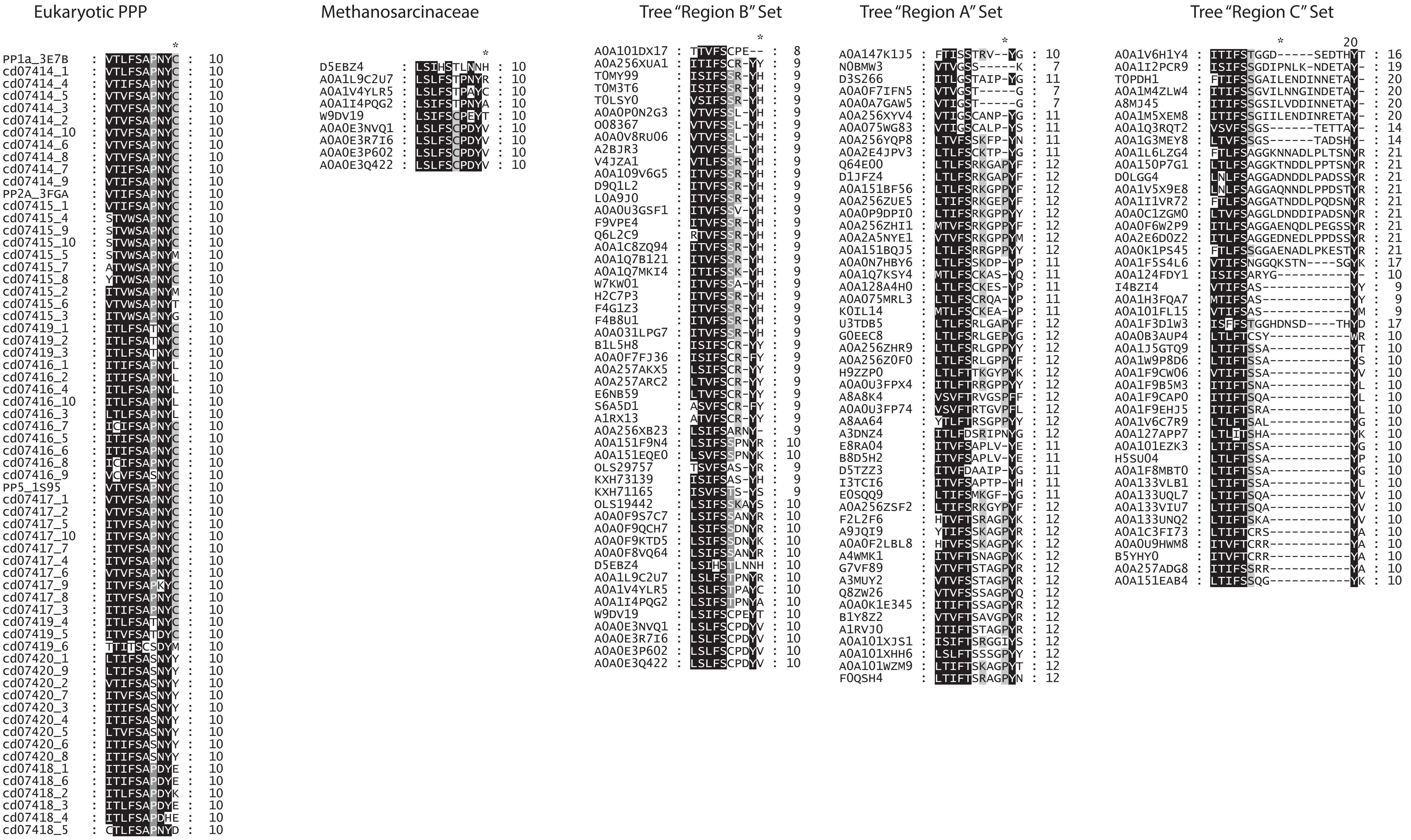

Supplemental Figure S9: Residue conservation in Motif 6 of PPP Sequences – Summary Alignments

Reference eukaryotic PPP sequences and candidate "EukaryoticPPP-Like" sequences from Archaea and Bacteria were collected, aligned, and phylogenetic trees constructed as detailed in Methods. Subalignments of the Motif 6 region were constructed as detailed in Methods. Graphical summary WebLogos were constructed as detailed in Methods for sequences in the major regions of the detailed phylogenetic tree of Supplemental Figure S7 and the radial phylogram of Figure 2 and presented together in Figure 3. This figure presents the conventional alignments which underlie each of those WebLogos. Sequences from solved structures are indicated by their 4-letter PDB codes. The database accession numbers and species of origin of cd07414, cd07415, cd07416, cd07417, cd07418, cd07419 and cd07420 sequences are given in Supplemental Table S5. All other sequence names are UniProt accession numbers and are listed in Supplemental Tables S1 - S3.

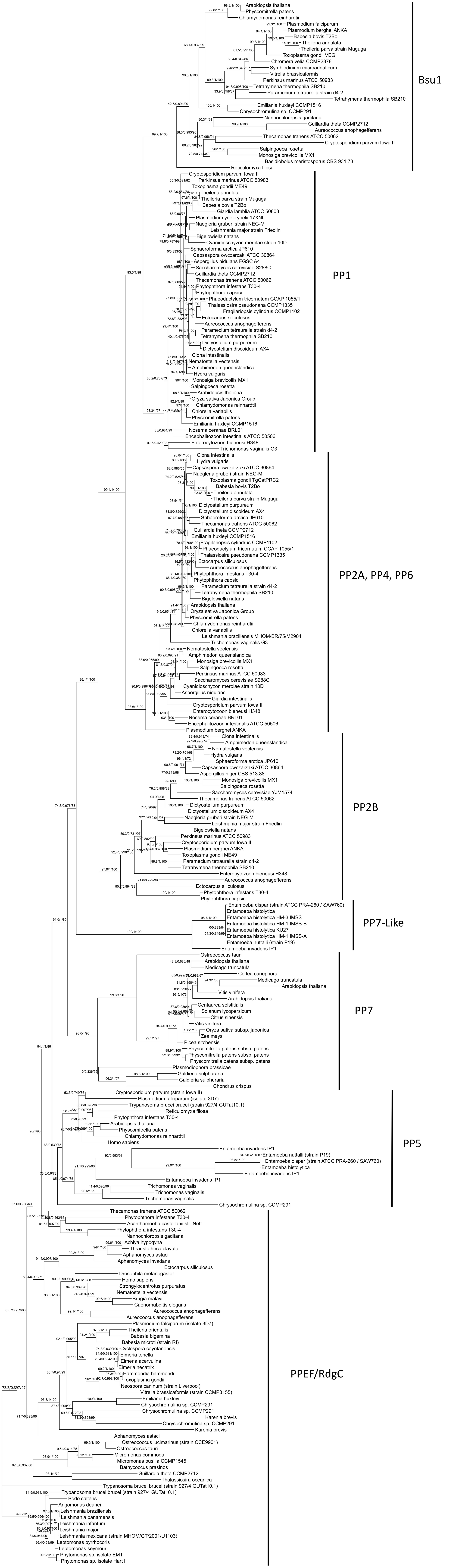

Bsu1
PP1
PP2A, PP4, PP6
PP2B
PP7-Like
PP7
PP5
PPEF/RdgC

Supplemental Figure S10: Eukaryotic PPP radiation – Maximum Likelihood phylogenetic tree

Reference eukaryotic PPP (phosphoprotein phosphatase) sequences were collected from the literature and from a search of a database from a panel of 45 completely sequenced eukaryotic genomes utilizing eukaryotic PPP HMMs (Hidden Markov Models) as detailed in Methods. Candidate novel sequences for eukaryotic PPP subtypes were collected from an iterative database search utilizing eukaryotic PPP HMMs as detailed in Methods. Sequences were aligned, and an unrooted Maximum Likelihood (ML) phylogenetic tree was inferred as detailed in Methods. Shown is an orthogonal phylogram. Eukaryotic PPP subclasses are indicated. The alignment giving rise to this tree is presented as Supplemental Figure S12. "PP7-Like" sequences are those sharing a common insertion with the classic PP7 group (discussed in the Text). Branch support values are: SH-aLRT/aBayes/UFBoot (see Methods). Details of the various sequences are presented in Supplemental Table S4.

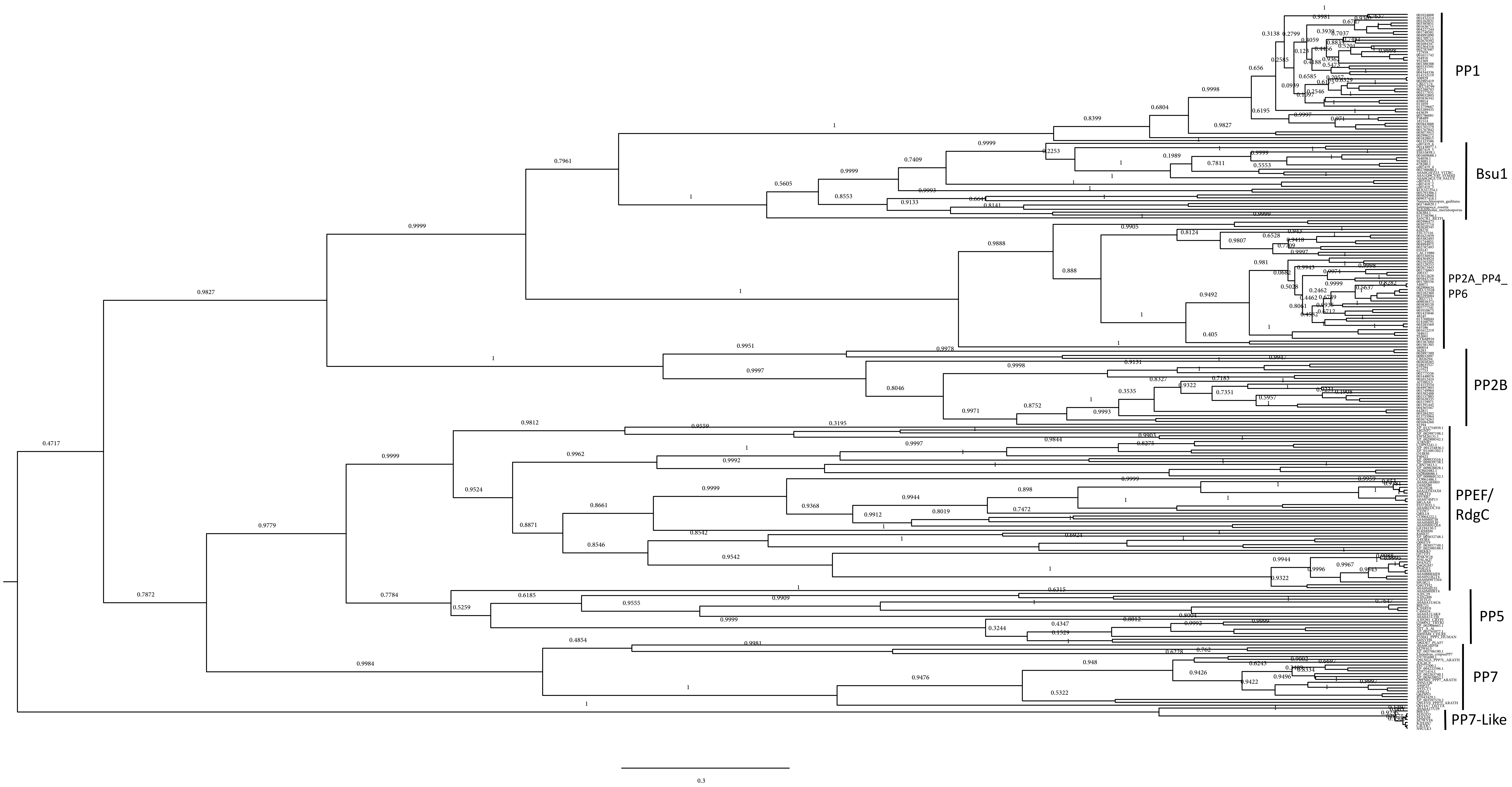
PP1
Bsu1
PP2A_PP4_
PP6
PP2B
PPEF/
RdgC
PP5
PP7
PP7-Like
0.3

Supplemental Figure S11: Eukaryotic PPP sequence radiation – rooted Bayesian phylogenetic tree

Eukaryotic PPP (Phosphoprotein phosphatase) sequences were collected, aligned, and a rooted Bayesian phylogenetic tree inferred by BEAST analysis as detailed in Methods. Shown is an orthogonal phylogram. Support values are posterior probabilities. Eukaryotic PPP subtypes are indicated. "PP7-Like" sequences are discussed in the Text. Details of the various sequences are presented in Supplemental Table S4.

Supplemental Figure S12

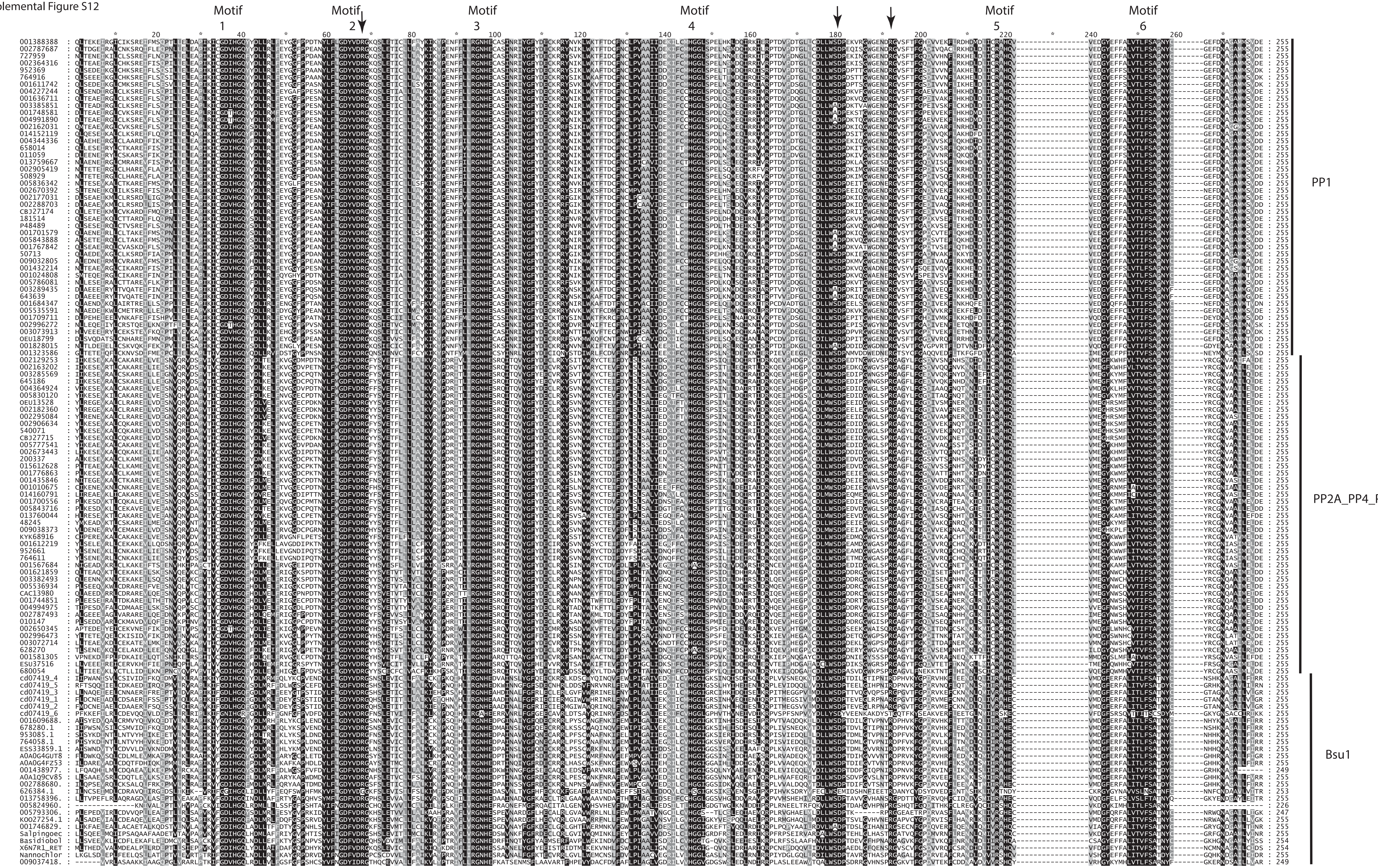

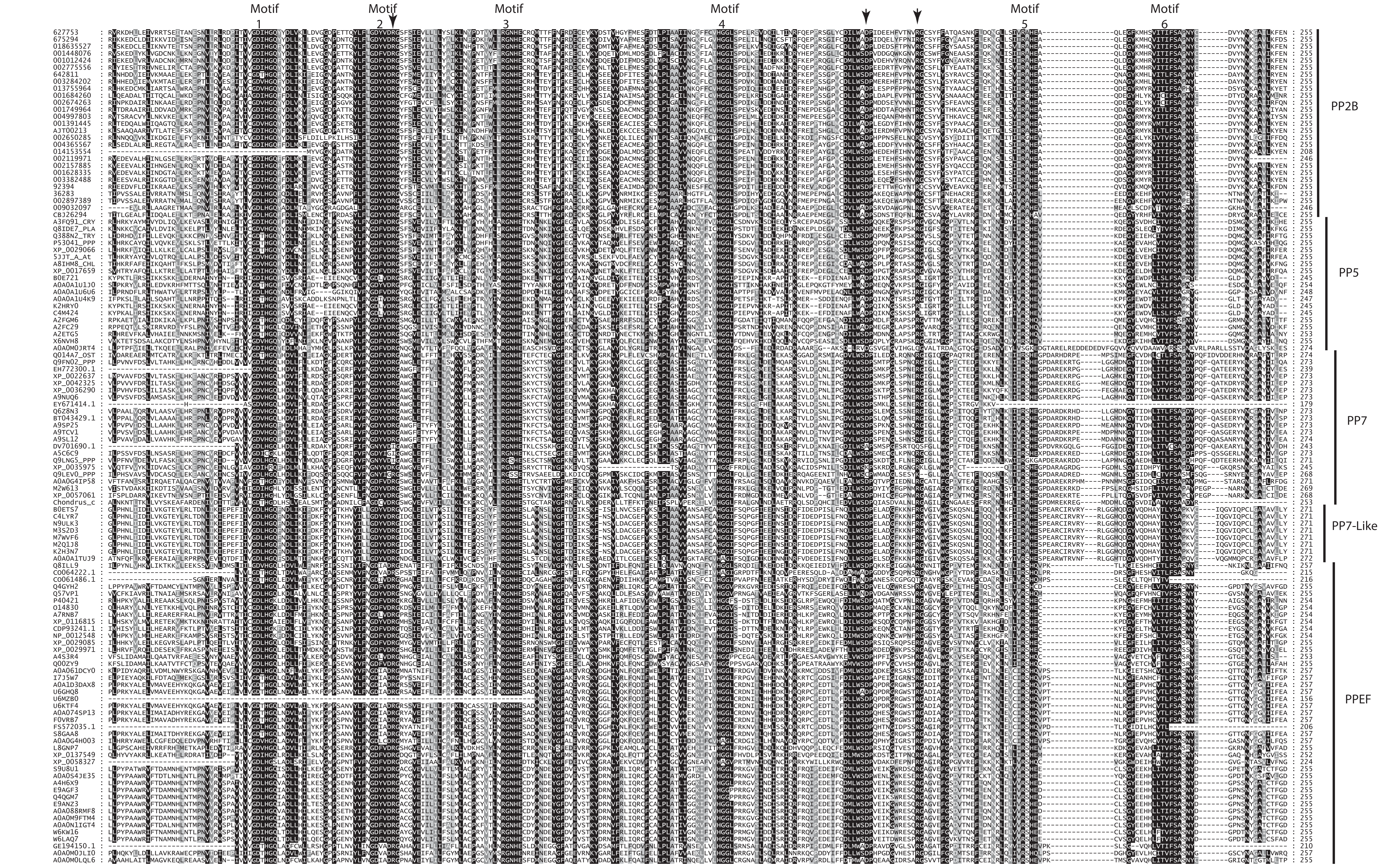

Motif 1
Motif 2
Motif 3
Motif 4
Motif 5
Motif 6
PP2B
PP5
PP7
PP7-Like
PPEF

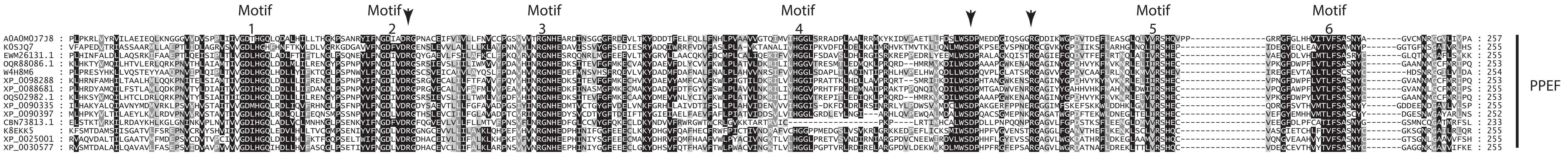

Supplemental Figure S12: Eukaryotic PPP radiation - alignment of reference and novel eukaryotic PPP sequences

Reference eukaryotic PPP (phosphoprotein phosphatase) sequences were collected from the literature and from a search of a database from a panel of 45 completely sequenced eukaryotic genomes utilizing eukaryotic PPP HMMs (Hidden Markov Models) as detailed in Methods. Candidate novel sequences for eukaryotic PPP subtypes were collected from an iterative database search utilizing eukaryotic PPP HMMs as detailed in Methods. Sequences were aligned as detailed in Methods. Conserved sequence motifs are indicated. The first and third arrows denote the first and second conserved Arg residues of the "2-Arginine Clamp". The second arrow denotes the Asp residue which in eukaryotic PPPs replaces the second conserved Arg of the bacterial PPP "2-Arginine Clamp". The phylogenetic trees resulting from this alignment are presented as Supplemental Figure S10 (Maximum Likelihood) and Supplemental Figure S11 (rooted Bayesian). The TCS (see Methods) score for this alignment is 975. Details of the various sequences are presented in Supplemental Table S4.

Supplemental Figure S13

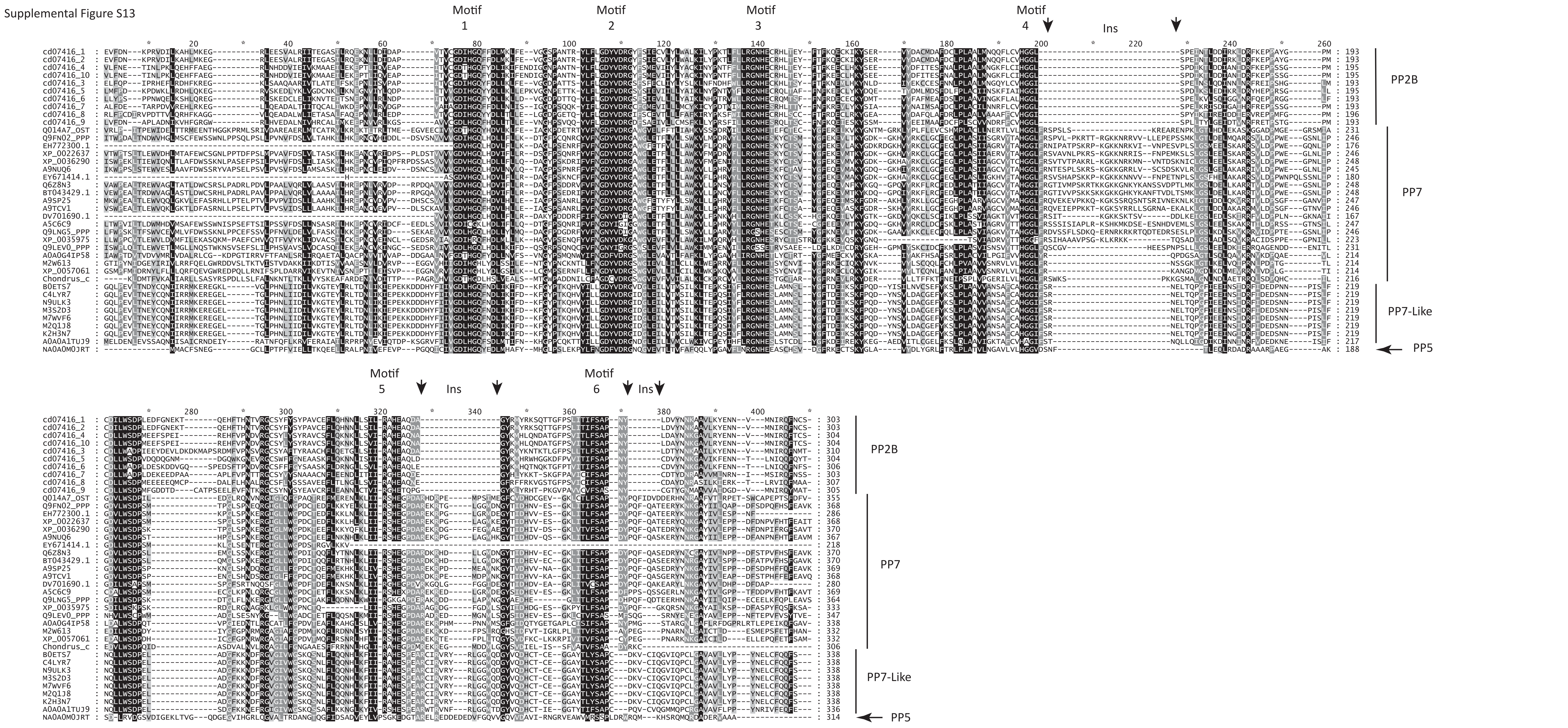

Supplemental Figure S13: Alignment of PP7 and related eukaryotic PPP sequences

Reference eukaryotic PPP (phosphoprotein phosphatase) sequences were collected from the literature and from a search of a database from a panel of 45 completely sequenced eukaryotic genomes utilizing eukaryotic PPP HMMs (Hidden Markov Models) as detailed in Methods. Candidate novel sequences for eukaryotic PPP subtypes were collected from an iterative database search utilizing eukaryotic PPP HMMs as detailed in Methods. Sequences were aligned as detailed in Methods. Conserved motifs are indicated. Regions between pairs of arrowheads ("Ins") are sequence insertions. Eukaryotic PPP subtypes are indicated. "PP7-Like" sequences are discussed in the Text. The TCS (see Methods) score of this alignment is 876. Sequence details are given in Supplemental Tables S4 and S5.

Supplemental Figure S14

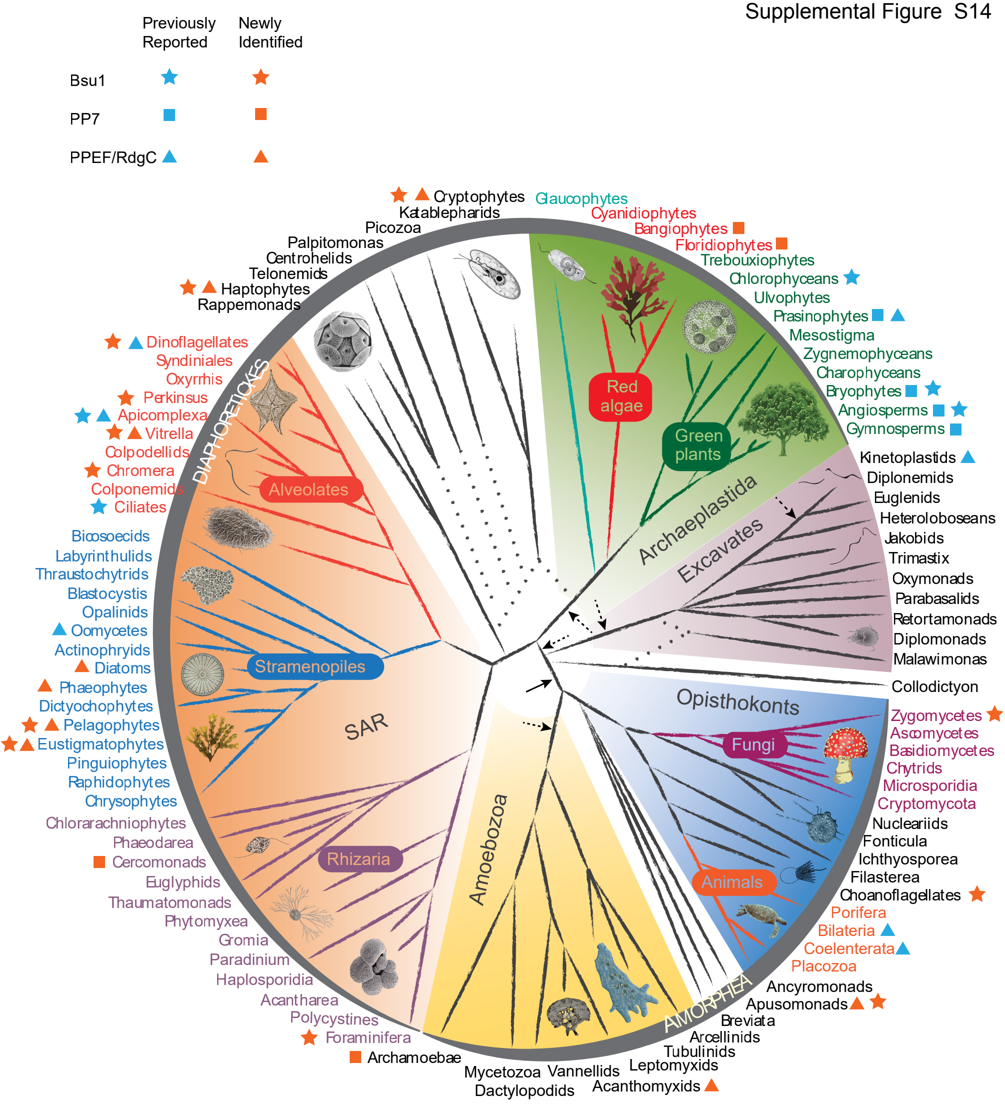

Supplemental Figure S14: Phylogenetic range of eukaryotic PPP subtype sequences. Candidate novel sequences for eukaryotic PPP (phosphoprotein phosphatase) subtypes were collected from an iterative database search utilizing eukaryotic PPP HMMs as detailed in Methods. Candidates were validated by sequence alignment and phylogenetic tree inference, as detailed in Methods. The alignment of these validated sequences, together with reference eukaryotic PPPs, is presented as Supplemental Figure S12. The phylogenetic trees encompassing these sequences are presented as Supplemental Figure S10 (Maximum Likelihood) and Supplemental Figure S11 (rooted Bayesian). The present Supplemental Figure is adapted, with permission, from a published report (see text for reference). It depicts a radial phylogenetic tree summarizing eukaryotic species diversity, with major organismal groups and their interrelationships indicated as defined in 2014 (burki ref). Peripheral blue symbols of different shapes represent the range of previously reported eukaryotic PPP sequences of various types. Orange symbols represent the novel eukaryotic PPP sequences of various types reported in this study.
